# A systematic review of the purposes of Blockchain and fog computing integration: classification and open issues

**DOI:** 10.1186/s13677-022-00353-y

**Published:** 2022-11-19

**Authors:** Yehia Ibrahim Alzoubi, Asif Gill, Alok Mishra

**Affiliations:** 1 American University of the Middle East, Al-Eqaila, Kuwait; 2 School of Software, University of Technology Sydney, Sydney, Australia; 3 Informatics and Digitalization Group, Molde University College-Specialized University in Logistics, Molde, Norway

**Keywords:** Blockchain, Cloud computing, Fog computing, Integration, Internet of things (IoT), Security

## Abstract

The fog computing concept was proposed to help cloud computing for the data processing of Internet of Things (IoT) applications. However, fog computing faces several challenges such as security, privacy, and storage. One way to address these challenges is to integrate blockchain with fog computing. There are several applications of blockchain-fog computing integration that have been proposed, recently, due to their lucrative benefits such as enhancing security and privacy. There is a need to systematically review and synthesize the literature on this topic of blockchain-fog computing integration. The purposes of integrating blockchain and fog computing were determined using a systematic literature review approach and tailored search criteria established from the research questions. In this research, 181 relevant papers were found and reviewed. The results showed that the authors proposed the combination of blockchain and fog computing for several purposes such as security, privacy, access control, and trust management. A lack of standards and laws may make it difficult for blockchain and fog computing to be integrated in the future, particularly in light of newly developed technologies like quantum computing and artificial intelligence. The findings of this paper serve as a resource for researchers and practitioners of blockchain-fog computing integration for future research and designs.

## Introduction

In 2012, Cisco introduced the concept of Fog Computing (FC) to improve network infrastructure to match the demands of the large amounts of data being transmitted to the cloud for processing [1]. That is, FC was introduced to help and overcome the problems faced by cloud computing (the use of the Internet to supply on-demand computer services such as storage, apps, and processing capabilities) such as the connectivity between the cloud and the Internet of Things (IoT) devices, the latency-sensitive applications, location awareness of the IoT applications, and complexity of the distribution environment [2–4]. FC is a cloud that is close to ground infrastructure, which is located near IoT devices to provide storage, connection, and control of IoT devices [5]. Therefore, the role of FC is to intermediate communication between the IoT devices and the cloud; however, it does not replace the cloud [6]. Moreover, FC enables and links IoT devices with services and on-demand apps [7, 8]. In addition, FC nodes assist IoT devices in carrying out computational operations that require higher power, which shortens the response time and fits the criteria of some applications that are time-sensitive on the IoT devices [9].

Because the FC is a cloud computing extension, it inherits several of the cloud’s issues [10]. The most notifiable issues are security and privacy [11], due to the resource-constrained capabilities of FC [12, 13]. Hence, to protect IoT devices, FC should provide effective solutions and techniques [14]. Although cloud computing has many techniques to mitigate the impact of security and privacy issues, these solutions may not be applied effectively for FC due to limited resources and unique characteristics such as decentralized structure, mobility (changing the users and the location of the fog node), and the different providers of the fog devices [14–16]. Therefore, FC requires new and innovative solutions to overcome security and privacy issues [11]. Moreover, FC is a resource-constrained system, which is reflected in the computation storage capabilities. This limitation has created another set of challenges related to the scalability of FC due to the ever-increased number of connected IoT devices. A BC’s capacity for processing transactions in mass quantity is referred to as scalability [17].

Blockchain (BC) technology has been dedicated to addressing the security and privacy of many applications [18]. It is also recommended to increase the storage capacity since it represents a distributed ledger [19]. BC is a decentralized technology architecture that originated from the use of digital encrypted currency (e.g., Bitcoin) [20]. Bitcoin is a well-known BC platform that contains an active cryptocurrency that enables distributed networks to conduct transactions without the use of middlemen or third parties [21]. BC technology is characterized by the capabilities of building reliable networks with no downtime as well as a high level of security and privacy [22]. BC has gained widespread attention from industries, governments, and financial institutions [23]. Several governments have included BC in their future informatization (e.g., China), called for the development of BC in their public sectors (i.e., USA), and started building BC pilot projects in core industries (e.g., South Korea) [24]. Recently, BC has started evolving as a significant structure for COVID-19 management in China. Chinese hospitals use BC technologies in several fields such as electronic health records, insurance claims, tracking of the supply chain, and identification of forged drugs [24].

As a result of this revolution of adopting BC, many papers have been, recently, published that devote BC as a potentially effective solution to address the issues of FC [19]. However, the literature in this domain is very diverse [25]. Although some papers have surveyed the previous literature; they lack critical evaluation criteria and methods for systematically reporting the results (e.g., [26]. Moreover, the majority of survey papers discussed one application (e.g., eHealth, IoT, vehicles, and so on) (e.g., [27–29]). To the best of the authors’ knowledge, the recent studies published in the public domain (at least, at the time that this study was initiated), lack a systematic review of the available literature about the purposes of integrating BC technology and FC. Additionally, this study addresses the purposes cited in all accessible literature from various applications, representing a thorough investigation. Hence, the main aim of this paper is to fill this literature gap and systematically investigate these purposes. Consequently, this paper focuses on the following research questions:

RQ1: How blockchain-fog computing integration purposes develop over time?

RQ2: What are the future challenges of blockchain-fog computing integration?

The main contributions of the paper are as follows. This paper provides the state-of-the-art purposes of BC-FC integration. This paper follows a critical evaluation of each reviewed paper by following well-defined and motivated criteria. This paper comprehensively reviews the work done so far in the field from different perspectives (e.g., algorithms, schemes, architecture, and so on). The literature on BC with FC integration is very miscellaneous; systematically organizing the relevant literature is a significant task [30]. Seven categories of the BC with FC integration purposes were identified; security, privacy, access control, trust management, data management, scalability, and performance. The paper also presents a roadmap of prospective research areas, problems, and possibilities for which more studies are required to guide the researchers. This was done by addressing the limitations of reviewed papers and identifying some open issues in infrastructure, platform, and technical limitations of BC architecture that distress processes in specific realms. It’s important to note that this analysis is by no means comprehensive since BC technology continues to advance at a breakneck speed. The rest of this paper is organized as follows. Research bacground presents an overview of BC. Blockchain overview discusses the research methodology. Blockchain with fog computing integration overview discusses the descriptive findings. Research methodology discusses BC with FC integration purposes. Locating studies discusses the future challenges and open questions about BC with FC integration. Study selection and evaluation concludes with options for further research.

## Research background

### Blockchain overview

BC can be defined as a distributed append-only public ledger technology that was originally proposed for cryptocurrencies (e.g., Bitcoin) [24]. In 2008, the concept of BC was proposed by [31]. Transactions occur among different parties without the supervision of a central authority. The valid transactions, using the consensus mechanism, are then recorded in the ledger (chronologically blocks that form a BC) and copied to all parties. A consensus algorithm is used to construct blocks and add them to the ledger which sometimes represents a computational issue. Three considerations are required for BC construction; immutable ledger, transparent and public ledger, and anonymity of the BC users [18].

The majority of the background body was built using bitcoin BC, which is the first and most widely used BC platform among a wide range of applications. Another reason for discussing Bitcoin BC in greater depth rather than other platforms such as Ethereum (a decentralized open-source BC with smart contract capability that is most recognized for its native cryptocurrency, ETH, ether, or just Ethereum) is the extensive literature accessible on the platform [32]. Bitcoin BC, for example, uses SHA-256 hashing and elliptic curve cryptography to provide robust cryptographic evidence for data integrity and authentication [20]. The elliptic curve cryptography is a key-based encryption system that employs pairs of private and public keys to encrypt and decrypt data [14]. The BC, usually, includes a list of all transactions and a hash to the prior block, which enables a cross-border distributed trust environment. While trusted parties or centralized authorities may misbehave and can be compromised, disrupted, or hacked, transactions in the public ledger of BC are validated by a majority consensus of miner nodes involved in the validation process [33]. In PoW-based BCs, for example, the validation occurs by calculating a hash with leading zeros to meet the difficulty target [20]. After validating by a consensus, the transaction data are saved in a ledger that not be erased or changed (data are immutable) [34].

**Fig. 1:**
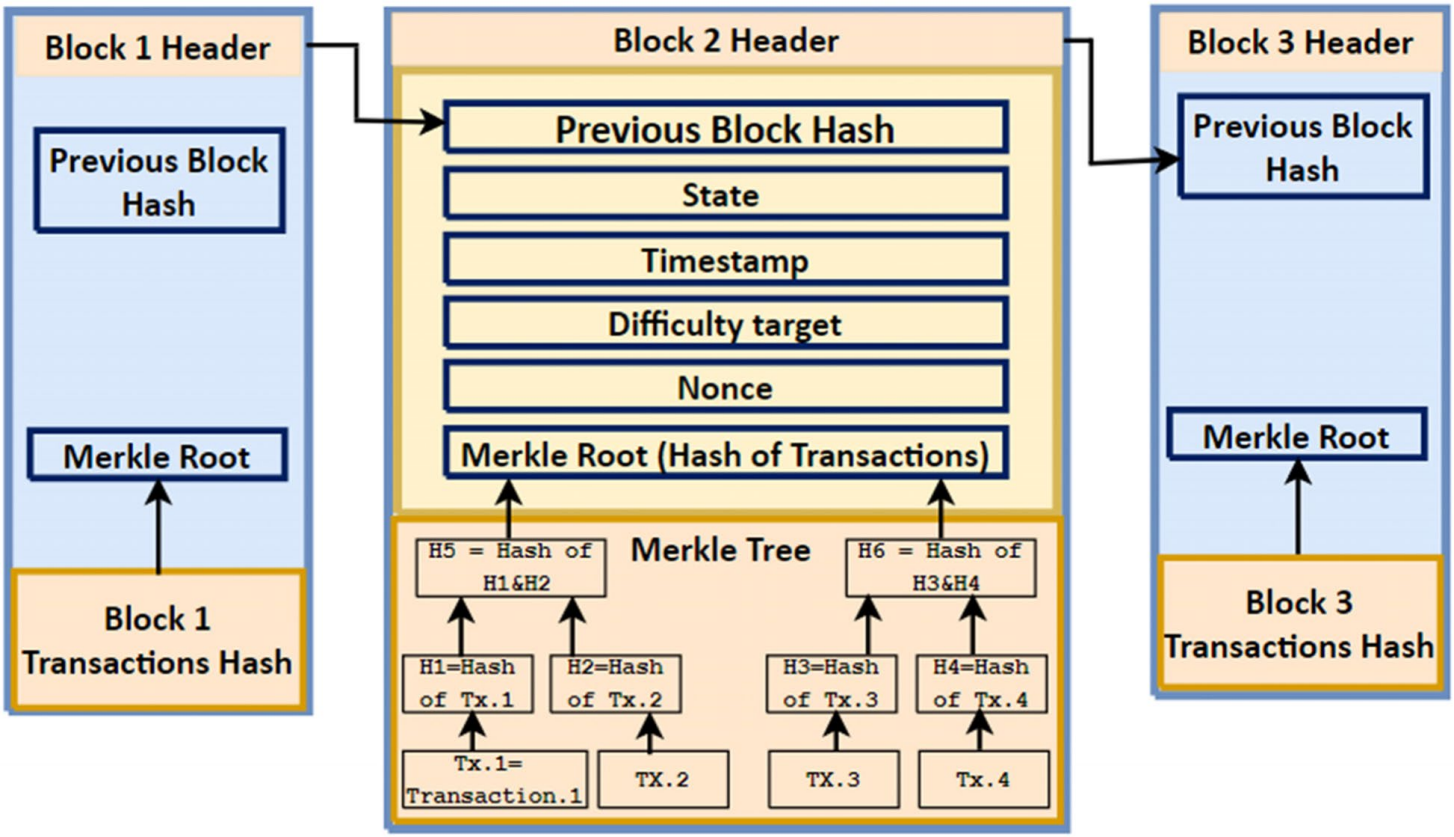
Bitcoin BC structure

Figure 1 describes a typical structure of the Bitcoin BC which consists of a sequence of blocks connected through the hash value. The BC includes the block header and the block body includes the transactions list. Various fields are included in the block header such as the block size, a timestamp, the number of transactions, and the version number. The hash value of the current block is represented by the Merkle root field. Hashing using the Merkle tree is often used in Peer-to-Peer (P2P) and distributed arrangements as it provides effective data proof. The nonce field is included as a Proof-of-Work (PoW) algorithm (the original consensus algorithm in BC (e.g., Bitcoin and Ethereum), which is used to confirm transactions and produce new blocks in the chain), and it is used to generate the trial counter value that generates the hash with leading zeros [32]. The number of leading zeros is specified by the difficulty target (i.e., used to preserve the block time of nearly 17.5 s for Ethereum and 10 min for Bitcoin [20]). The difficulty target can be modified to increase the number of zeros if the computation power of the hardware increased. The timestamp is used for tracking the modification on the BC. Different mechanisms are used for timestamping such as signing using the private key of a trustworthy server used in the traditional schemes [35]. Another technique can be used by deploying distributed timestamping which helps to avoid a single point of failure [35].

The method by which a BC network achieves consensus is referred to as a consensus mechanism or algorithm. Since there is no central authority, the public BC (i.e., decentralized) is constructed as a distributed mechanism, with distributed nodes agreeing on the validity of transactions using a consensus algorithm [34]. In other words, BC depends on distributed consensus to validate the transactions which guarantee the consistency and integrity of the transactions [36]. The different consensus mechanisms impact the BC system differently [37]. The best (idealistic) consensus mechanism promotes giving the same weight to all miners for the validation process and then deciding based on the majority. This ideal scenario may be applicable in a controlled (private) environment; however, in public contexts, this may increase the chance of Sybil attacks as users can share multiple identities [35]. In distributed architecture such as FC, only one random user will add every block which may lead to several attacks [38].

Bitcoin is the most well-known cryptocurrency. Later, in 2015, Ethereum BC was launched, which can execute smart contracts and store data [38]. The smart contracts are programs written and uploaded by parties to be executed in the BC which includes the terms of the contract. Soon later, other BC platforms were launched such as Stellar (a digital money protocol that’s distributed and open-source), Hyperledger (a worldwide business BC initiative that provides the structure, tools, and rules for creating open-source BCs and apps), Ripple (a BC-based digital payment system and mechanism with its cryptocurrency, XRP), Eris (an open-source software that enables anybody to create low-cost, safe, and portable apps utilizing smart contract and BC technology), and Tendermint (an algorithm for securely and consistently replicating applications over many devices) [20, 21, 32]. Depending on the data managed, the availability of that data, and the actions taken, different types of BC can be identified. It is worth mentioning here that some authors refer to public/permissionless and private/permissioned, interchangeably. This can be applicable in cryptocurrencies; however, in other applications that need to distinguish between authentication and authorization, it’s not applicable. Though, the naming is still in debate among authors. Note that Bitcoin, for instance, is used to track digital assets, while smart contracts used in Ethereum enable certain logic. Moreover, while some system like Ripple makes use of tokens, others like Hyperledger do not.

In general, BC can be categorized into three major types; public (e.g., Bitcoin, NXT, CounterParty, RootStock, and Zcash platforms), private (e.g., Monax, Hyperledger Fabric, Ripple, Multichain, and Corda platforms), and consortium (e.g., Ethereum, Monax, and Multichain platforms) [23, 24, 39]. 1) On the Internet, everyone can see the public BC ledgers, and anybody may validate and contribute a transaction to the BC. 2) Only selected people inside the company may add and validate transactions in Private BC, but anybody with access to the Internet can normally read them. 3) Consortium BC allows only a group of organizations (e.g., financial institutes) to add and validate a transaction, however, the ledger might be available or limited to certain parties. Applications such as auditing within an organization and data management require consortium BC, in general, as public BC is not suitable for user privacy and commercial benefit protection [23].

Accordingly, BC offers the following benefits over other technologies [34, 36]. 1) Resilient - no single point of failure and using smart contracts, which means BC helps in transferring, securely. It is a network of nodes, all nodes work collaboratively to maintain the transaction, records are augmented to a ledger of a previous transaction, and PoW should be validated by other nodes included in the chain. 2) Decentralized and trustless-P2P system, which cuts the need for any kind of agent for security by cryptography. The distributed database is duplicated into every node, which includes timestamps, transaction lists, and information with links to the previous blocks in the chain. The distributed ledger should be transparent, immutable, publicly accessible, and updated after each transaction. 3) Scalable and high speed and capacity technology. The computing capacities of the network scale up when a new peer joins the chain. 4) Secure and transparent because every transaction is visible to every miner on the chain.

While a lot of research has been conducted on BC technology, the state-of-the-art of BC with FC integration purposes has received insufficient attention [40]. The main impetus for this work was the lack of a clear and complete analysis of existing BC with FC integration purposes state-of-the-art in the literature. BC can avoid many attacks even without centralized control or data storage [23]. The Ethereum-transaction-based state-machine provides special features like security, transactional privacy, integrity, authorization, auditability, data immutability, fault tolerance, and transparency [24]. Accordingly, many applications use this technology nowadays rather than cryptocurrencies such as smart transportation, identity management, industry, agriculture, energy grids, supply chain management, and FC [22].

### Blockchain with fog computing integration overview

FC is a highly dispersed computing structure with a set of assets made up of one or more pervasively linked embedded systems (which include IoT devices) supported by cloud computing, to cooperatively offer storage, computation, storage, connectivity, and other services to a sizable number of IoT devices nearby [3]. FC is a cloud expansion that is more closely connected to IoT devices. FC serves as a bridge between edge devices (e.g., sensors, and actuators) and the cloud [14]. A fog node could be any device having processing power, storage capabilities, and network connection, including routers, security cameras, switches, and control devices. Distribution, flexibility, proximity to IoT devices, low latency, real-time transactions and analysis, and heterogeneity are typical characteristics of FC [41]. All of these qualities made FC a very alluring remedy for cloud computing problems, particularly excessive latency and centralized authority [42].

Many studies have been conducted recently that discussed the value of BC in an FC environment such that devices like personal computers, mobile units, and Vehicular Ad-hoc Network (VANET) can be equipped with BC. The role of BC in FC can be broadly seen from two angles; data processing and communication [43]. That is, the role of BC will be very important in maintaining security and privacy on the fog nodes when data is stored or processes in the fog node and when data is transferred between fog nodes, between fog nodes and the cloud, and between fog nodes and the IoT devices. The fog node will play the operator role (i.e., manage) for IoT devices [14]. The decentralized and dispersed fog nodes, associated with the network, handle the communications included in BC. Each block in the BC is attached to the chain sequentially [34]. All nodes included in the BC environment are parts of the network which store a local copy of the transaction data permanently. All the parties involved jointly authenticate the transaction to meet a consensus decision, before a miner node (e.g., Ethereum Virtual Machines - nodes that can provide trustworthy execution cryptographically tamper-proof and administration to these contracts or programs) add the validated transaction into a timestamped block [20]. And then broadcasts it into the network. This data is periodically updated among all nodes for consistency purposes. This enables many nodes, that do not trust each other, to achieve authentication decisions based on the old transactions. In the BC environment, a public ledger preserves the validated transactions in a P2P network. In general, two keys are used: 1) a private key which is used to sign the BC transaction before broadcasting to other peers and 2) a public key that represents the unique address [18].

In order to obtain BC incentives, nodes compete in PoW to perform cryptographic formulas and verify transactions. On the other hand, Proof-of-Stake (PoS) employs random selection validators to guarantee the transaction’s dependability and pays them with cryptocurrency [44]. The most popular cryptocurrency, Bitcoin, employs PoW. The second-largest cryptocurrency, Ethereum, began off with PoW but is now switching to PoS. High levels of reliability and security are stated for PoW [45]. The intricacy of the mathematical calculations required to attain verification makes manipulating the system all but useless. But it’s slow and expensive to run, and it consumes a lot of energy. PoS eliminates the need for difficult calculations. Instead of figuring out a numerical riddle, the miner in PoS-based BC employs a digital signature as evidence. Instead of receiving a newly formed asset, the miner who verifies the block is compensated with a transaction fee [46]. PoS consensus maintains the incentive mechanism and effectively assures node equity since it has a low relative burden on computational resources and high throughput. By examining the quantity and duration of tokens it has, PoS calculates the likelihood of acquiring accounting privileges [47]. Similar to the stock dividend system, people who possess comparatively greater shares might get higher dividends. Therefore, it is more energy-efficient than PoW and provides higher sustainability [48]. The nodes with stakes are meant to be trustworthy and refrain from manipulating transactions, but if they do, their stake might be taken away. Participating in the PoS is simpler for investors than the PoW since it doesn’t need technical skills or computer-aided design. PoS outperforms PoW in terms of speed as well. For instance, Ethereum can handle up to 100,000 transactions per second using PoS, but it can only handle 30 transactions per second with PoW [48]. In the case of PoS, however, there is a possibility that a node will not have enough assets, in which case, if it were to be chosen as a miner, it would be viewed as malicious since it would have no assets to be debited [47].

## Research methodology

To identify and synthesize the purposes of integrating BC in FC, we adopted a Systematic Literature Review (SLR) approach based on the guidelines provided by [49, 50]. SLR aims to identify, select, and synthesize the available literature to answer the research question [30]. A systematic literature review protocol is essential to guide the review process [30] that provides a framework to understand the impact of BC on FC security and privacy challenges. We have developed a review protocol to validate the classification process of this paper. Distinct stages have been applied: (1) locating studies, (2) screening studies, (3) study selection and evaluation, and (4) study inclusion.

### Locating studies

The following seven well-known electronic databases were used in this review. These databases are expected to provide enough literature coverage for this paper.

- IEEE Xplore (www.ieeexplore.ieee.org/Xplore/).
- ACM Digital Library (www.portal.acm.org/dl.cfm).
- Elsevier ScienceDirect (www.sceincedirect.com/).
- SpringerLink (www.springerlink.com/).
- Google Scholar (http://scholar.google.com.au/).
- Emerald Insight (https://www.emerald.com/insight/).
- Wiley Online Library (https://onlinelibrary.wiley.com/).
- SAGE Publication (https://us.sagepub.com/en-us/nam/home).
- MDPI Online (https://www.mdpi.com/journal).

**Fig. 2:**
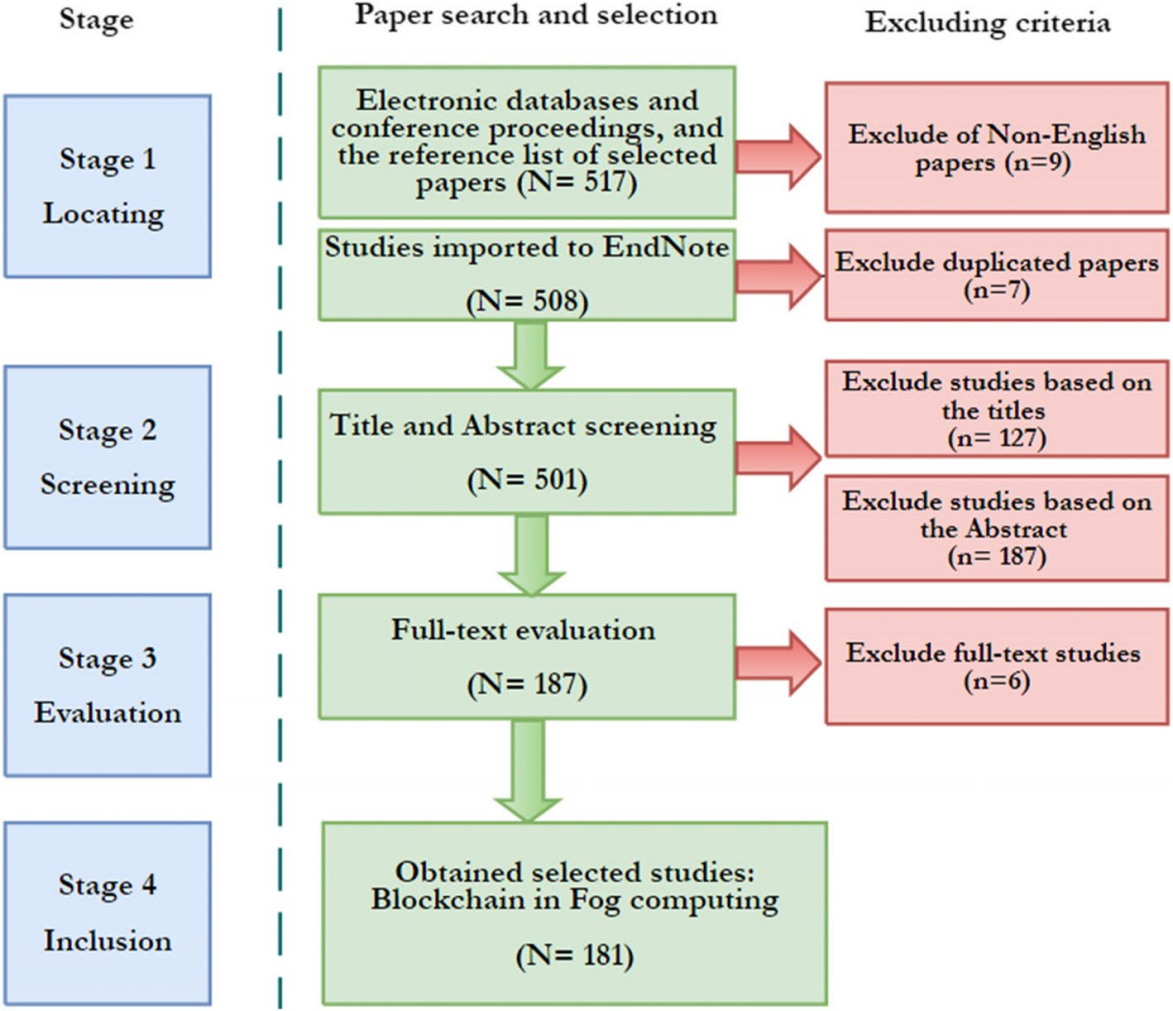
Study selection process

In the first stage, all possible combinations of BC, FC, and edge computing were searched using the Boolean “AND” and “OR” operators. The edge computing term was included in the search terms because many authors refer to FC as edge computing. The selected studies come from different IoT applications of FC such as vehicular, smart cities, and health applications. The selected papers include peer-reviewed articles published in journals, book sections, or conference proceedings. Figure 2 shows the stages of the review process and the number of papers identified at each stage. In this review, we included any study that discussed BC as a technique used in fog or edge computing. Therefore, studies were excluded if their focus was not on fog or edge computing or if they did not discuss using BC. This review included studies up to April 2022; qualitative, quantitative, mixed measurement studies, overview studies, and review studies. The search excluded studies that discuss prefaces, poster sessions, editorial discussion, news, article summaries, or reader’s letters. Only papers written in English were included.

### Study selection and evaluation

The authors individually evaluated all of the literature using the established criteria, as discussed in Section 3.1. All authors sat together, at the end of each stage, and discussed the included and excluded studies. In this review, we followed the citation procedure discussed in Alzoubi et al. [50]. We used EndNote as a citation manager tool to store the selected studies. Moreover, we used the backward snowball sampling technique and searched the reference lists of the selected studies, in the first stage, to get new studies. The number of hits resulting from the first stage was 517. After excluding the non-English written studies, the number dropped to 508. Moreover, the number dropped to 501 after excluding the duplicated papers.

The 501 papers were imported to EndNote (to keep track of the references) and Excel sheet (to maintain the abstracts and titles). In this stage, the titles of the selected studies were reviewed. The papers that were not about BC with FC integration were excluded. However, some titles failed to be identified, and thus included in the next review stage. In this stage, 374 studies were identified as relevant to the scope of this study. Moreover, after reviewing the abstracts of the selected papers, the number dropped down to 187 papers. The abstracts that were not considering any application of BC with FC integration were excluded (e.g., architectural and/or technological features of BC). Some abstracts were misleading so the papers, in this case, were included in the next stage. If the abstract was not available, the study was left for stage 4. At stage 4, all potential studies were gone under the full-text review. In this stage, 6 papers were excluded as they did not report the BC with FC integration, leaving 181 papers for the final inclusion stage.

### Data extraction and synthesis

All articles that matched the requirements for inclusion were entered into MAXQDA11, a qualitative analysis program, and the data was evaluated for emergent themes. The thematic analysis for selected papers was conducted independently by the authors. In the end, the seven categories were compared among all authors. The consensus rate was around 78%. All authors agreed on all articles included for thematic analysis (*N* = 181), one set of categories, and sub-categories. The selected studies [38–218] are discussed in the following Sections. First, a descriptive analysis is provided for the selected studies. Next, the taxonomy of the BC with FC integration’s positive impact on security and privacy issues of FC is presented. Finally, the future directions of this SLR are discussed.

## Descriptive analysis

The study looks at 181 academic articles that were published between 2016 and April-2022. The descriptive analysis serves several aims including fascinating insights into current research patterns in BC technology. It also serves as a guide for future studies. Moreover, it aids in visualizing the interdisciplinary research techniques that have been established in the scientific literature thus far. Table 1 summarizes the studies that were chosen based on the published database. IEEE was the single largest publication outlet, with 104 studies out of 181 (74 journal articles and 30 conference proceedings), followed by Elsevier Science Direct with 21 research. As the smallest number of studies, just two were retrieved from SAGE. The “IEEE Access” journal, which published 19 papers, was found to be the single most popular publication channel. Table 1 further reveals that the bulk of the papers chosen (132 out of 181) were peer-reviewed journal articles, followed by 43 conference proceedings, and only six-book sections.

**Table 1:** Publication channel

Database	Publication type	Study	Number
IEEE Xplore Total = 104 (57.4%)	Journal	[27, 48, 51–122]	74
	Conference Proceedings	[43, 123–151]	30
Elsevier Science Direct Total = 21 (11.6%)	Journal	[152–172]	21
Google Scholar Total = 15 (8.3%)	Journal	[173–180]	8
	Conference Proceedings	[181–186]	6
	Book Section	[187]	1
SpringerLink Total = 13 (7.2%)	Journal	[188–192]	5
	Conference Proceedings	[193–195]	3
	Book Section	[196–200]	5
MDPI Total = 12 (6.6%)	Journal	[201–212]	12
Wiley Online Library Total = 9 (5%)	Journal	[213–221]	9
ACM Digital Library Total = 5 (2.8%)	Journal	[222]	1
	Conference Proceedings	[223–226]	4
SAGE Publication Total = 2 (1.1%)	Journal	[227, 228]	2

**Fig. 3:**
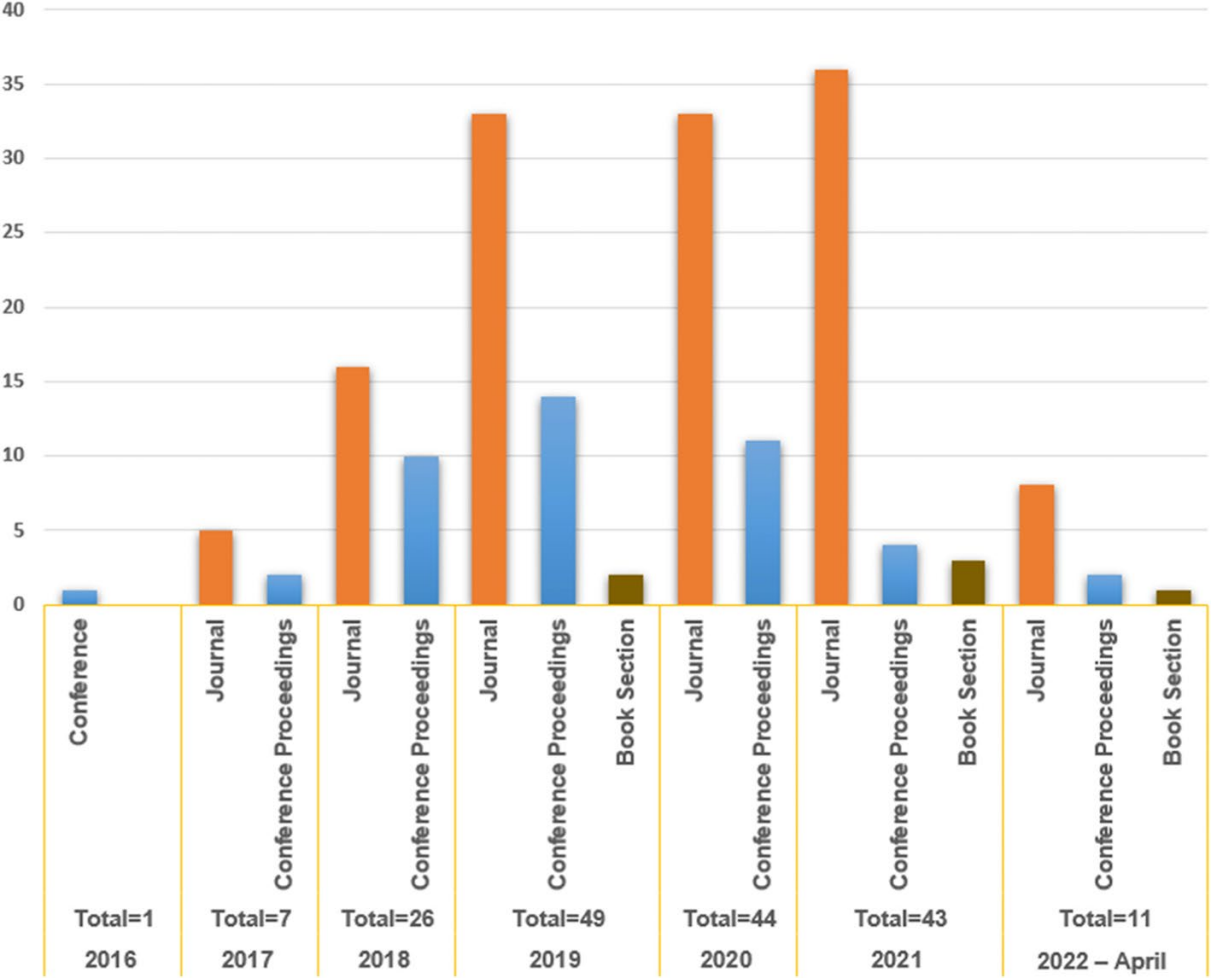
Publication year-distribution

Figure 3 shows a year-by-year examination of the selected publications. It’s worth mentioning that the number of publications was low in 2016 (1 study) and 2017 (7 studies), but increased in 2018 to 26 studies. However, in 2019, the total number of studies hit a high of 49. The number drops to 44 studies in 2020, 43 studies in 2021, and 11 studies by April 2022. This trend reflects the fact that BC technology is new and developing, as well as the increasing scholarly interest in it. Even though BC technology was initially established using Bitcoin as a basic underlying innovation and Bitcoin has accounted for the majority of investigated platforms over the last seven years, many of the papers published in 2020, 2021, and 2022 focused on the latest or modern BC platforms, such as Etherium, with a particular focus on smart contracts. Figure 3 also shows that the vast bulk of the literature was published in peer-reviewed journals, with only a few book sections.

BC originally started with Bitcoin (BC 1.0), then BC 2.0 which was built on smart contracts, and later evolved into coordinative applications (BC 3.0) [35]. The majority of BC with FC integration applications discussed in selected studies were IoT applications (83 studies), transportation (31 studies), eHealth (16 studies), industrial IoT (9 studies), monitoring applications (6 studies), energy (8 studies), mobile devices (4 studies), supply chain management (4 studies), drones’ network (3 studies), video streaming (2 studies), financial (2 studies), global collaboration (2 studies). Other applications were also revealed including FC-PoW approach [45], higher education applications [207], FC-resource brokerage platform [134], FC-authentication scheme [218], agricultural supply chain [156], multi-party contract signing [217], video streaming [99], consensus for edge-centric IoT [57], intelligent and safe task offloading in vehicles [89], and FC-rogue nodes approach [135]. Other review papers focused on BC with FC integration in general ([63, 85, 109]) and FC security ([45, 200]). The other studies which included literature review are not counted in these applications.

**Fig. 4:**
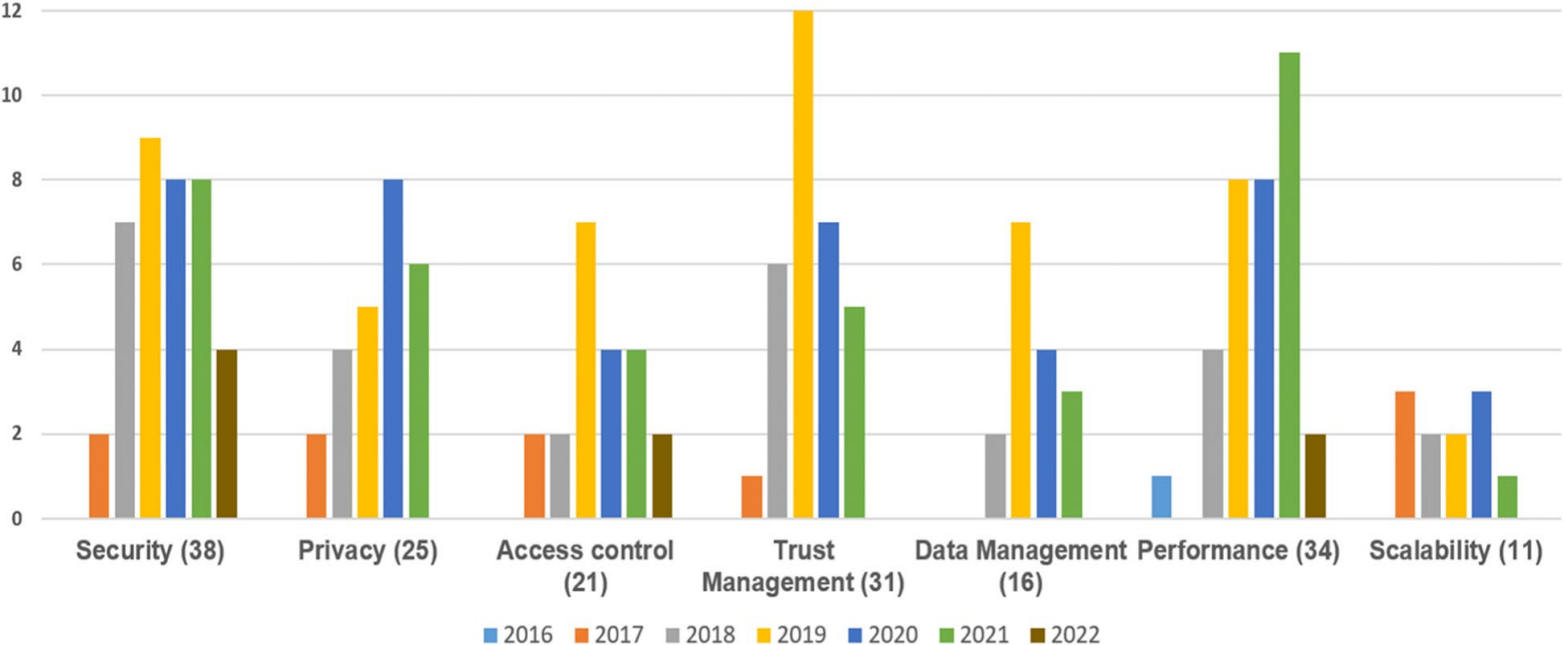
Domain year-distribution

Figure 4 shows the domain-purpose distribution of the 181 research across time. BC with FC integration has been divided into seven domains based on the results of the research. Security takes up the most research items (38 out of 181), followed by performance (34 studies), trust management (31 studies), privacy (25 studies), access control (21 studies), data management (16 studies), and lastly scalability (11 studies). Figure 4 demonstrates that, even though BC with FC integration is still in the early phases, its goals have expanded beyond security and privacy to include trust management, data management, performance, and scalability concerns. Furthermore, a significant number of publications addressing the subject of trust management were published in 2019 (12 studies). Moreover, the focus among the selected studies has been more on enhancing FC-IoT-cloud architecture using BC technology. It’s worth noting that several authors highlighted the role of BC in FC as a supplement to security and privacy concerns. In other words, they assumed that, by default, BC enhances the security and privacy of FC, then can achieve other purposes such as trust management, performance, or scalability. As a result, when classifying the results in this paper, we focused on the primary goal of each study.

Twenty two papers were literature review includes one paper published in 2016 ([125]), one paper in 2017 ([57]), two papers in 2018 ([63, 185]), seven papers in 2019 ([27, 109, 164, 194, 207, 210, 228]), three papers in 2020 ([85, 86, 195]), five papers in 2021 ([60, 191, 192, 200, 211]), and three papers up to April 2022 [153, 154, 214]. Some of these papers focused on certain purposes such as resource management [154], while others were general literature reviews without focusing on certain purposes such as [214]. The latter, though, were not included in the classification of Fig. 4.

More information about the survey studies found in our systematic evaluation is provided in Table 2. While the bulk of these surveys concentrated solely on a single area of BC with FC integration, such as health or transportation, or a single purpose, such as security and privacy, this article offered a thorough analysis of all purposes and from all areas of literature that were accessible. Furthermore, unlike this article, none of the identified survey studies have systematically investigated BC with FC integration.

**Table 2:** Survey studies focus

Study	Year	Focus
Samaniego et al. [125]	2016	Survey on integrating BC and IoT networks
Yeow et al. [57]	2017	Review of the decentralized consensus systems for edge-centric IoT
Uriarte & DeNicola [63]	2018	Survey on integrating BC and cloud/FC solutions
Pahl et al. [185]		Survey on BC platforms for IoT-edge computing
Abdulkareem et al. [27]	2019	Survey on FC and machine learning
Fernández-Caramés & Fraga-Lamas [207]		Survey on BC, IoT, FC and edge computing in universities campuses
Iqbal et al. [228]		Survey on BC, FC, and trust management in social Internet of vehicles
McGhin et al. [164]		Survey on BC in healthcare applications
Podsevalov et al. [194]		Survey on integrating BC and FC platform
Tariq et al. [210]		Survey on security of big data in FC-IoT applications and BC
Yang et al. [109]		Survey on integrating BC and edge computing
Baniata & Kertesz [85]	2020	Survey on integrating BC and FC
Bhattacharya et al. [195]		Survey on BC and edge computing
Ferrag et al. [86]		Survey on integrating BC protocols for the IoT networks
Aloqaily et al. [60]	2021	Survey on BC for 5G-UAV networks
Du et al. [211]		Survey on integrating BC-edge for IoT networks
Kiwelekar et al. [200]		Survey on integrating BC and FC for security
Liu et al. [191]		Survey on integrating BC-based resource allocation for edge computing in IoT applications
Mikavica et al. [192]		Survey on BC security, privacy, and trust management in vehicular networks
Deepa et al. [153]	2022	Survey on using BC for big data analysis
Kamruzzaman et al. [214]		Survey on integrating BC and FC for IoT healthcare services in smart cities
Hamdi et al. [154]		Survey on using BC for task offloading in vehicular FC
This survey		Systematic review of all potential integration purposes for FC and BC till April 2022

## Blockchain-fog computing purposes

**Fig. 5:**
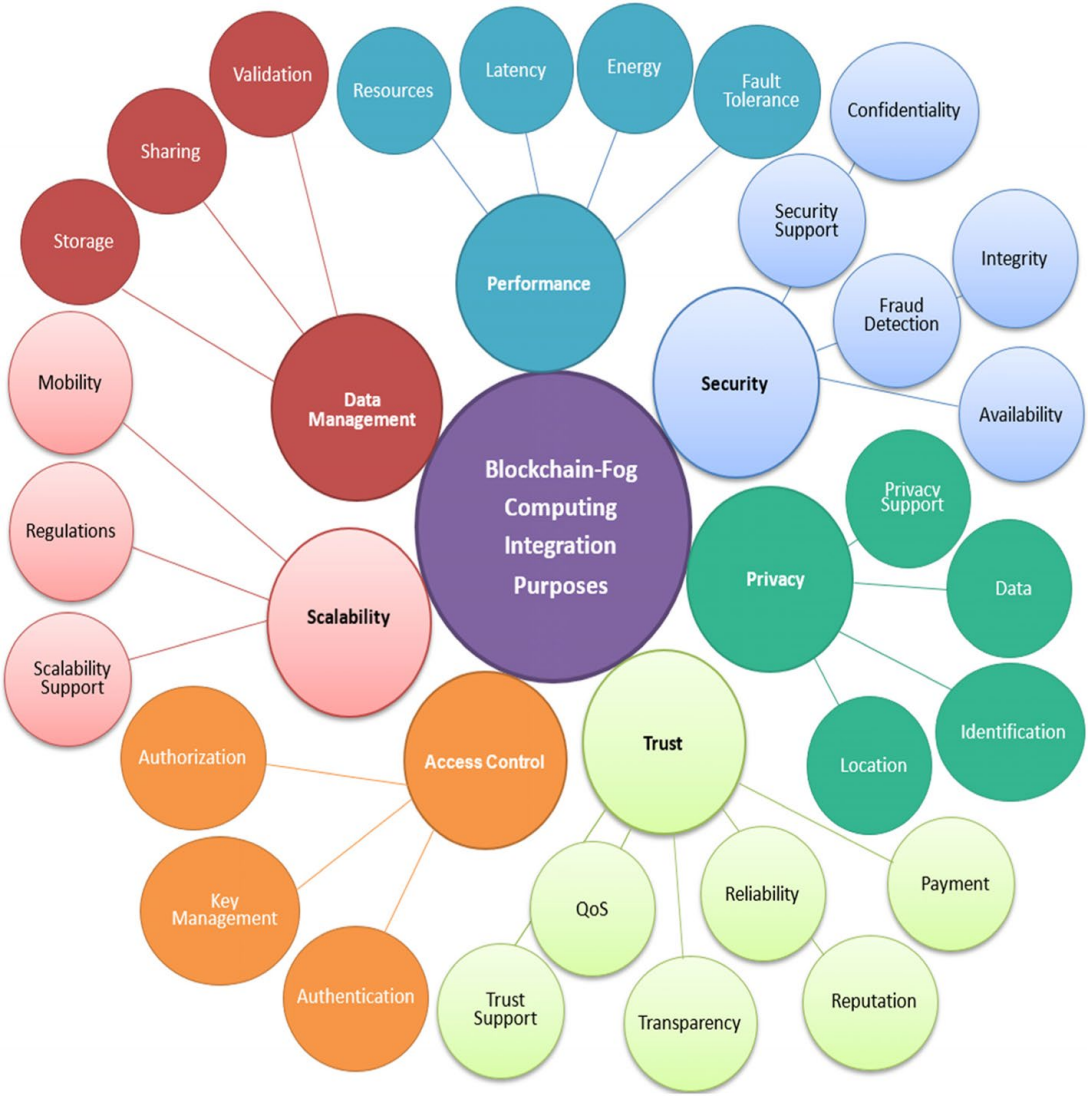
BC with FC integration purposes

This paper focuses on BC with FC integration purposes. We suggest a purpose-oriented categorization in this paper. Our approach, on the other hand, varies from comparable studies (E.g., [154, 192, 211, 214]) as it does so by utilizing a rigorous statistical methodology based on the literature, making it more relevant to present BC advances and illustrating future BC trends with high fidelity. As a result, we present a thorough and comprehensive classification of BC-based goals, which is visually depicted in Fig. 5, taking into consideration the current and future variety of BC solutions. Based on an examination of the existing literature, we provide a thorough taxonomy of the BC-enabled purposes that are currently accessible in the following subsections. The purpose categories identified in this paper were, however, classified (coded) using the prior literature review publications as a starting point. Most research reviews, for example, identified security and privacy as the major purposes of BC with FC integration. We begin classifying with these purposes and then add the evolved categories like trust, performance, access control, and scalability. The coding was done according to the definitions given to describe each purpose category and its subcategories.

### Security

Data can be harmed by a variety of security risks. BC may be able to shield you against these dangers to a large extent. Availability, confidentiality, and integrity are the most important security purposes [192]. We found several studies that indicated security support and fraud detection in addition to these three purposes. These purposes are discussed in the following sub-sections.

#### Security support

Many studies have reported that BC can enhance and support the security of FC, in general without focusing on a specific particular of security. Several new solutions were proposed to enhance security in the BC with FC integration environment. To provide an efficient and secure communication framework, Alam [176] emphasized the confluence of BC, FC, and IoT technology advancements. Similarly, Alam [177] presented a framework for delivering middleware on the Internet of smart devices network. The suggested framework is particularly well suited to applications in which data is sent regularly on the Internet of smart devices environment. Ashik et al. [139] created a FC-cloud architecture based on BC that may be utilized in smart homes. By leveraging the BC network, this design gives rise to a distinct fog architecture that provides greater security against known threats to safeguard our sensitive data. Dorri et al. [82] suggested a BC-based architecture to safeguard users’ privacy and strengthen the vehicular ecosystem’s security.

Huang et al. [81] proposed a distributed security approach using smart contracts and the lightning network; this suggested model is known as the lightning network and smart contracts. To improve the security of trade between charging piles and electric cars, the new suggested security model can be combined with existing scheduling software. Huang et al. [84] proposed a BC system to address the IIoT security problems. The authors also created a data authority management technique to control access to sensor data to safeguard sensitive data confidentially. Huang et al. [94] used BC technology to create a decentralized parked vehicle aided FC. Smart contract executions arrange and validate request posting, workload completion, task appraisal, and reward assignment automatically. This method provides strong security and efficiency guarantees, as demonstrated by a security study and comprehensive numerical findings [94].

Rahman et al. [104] demonstrated a safe therapeutic framework that allows patients to own and control their personal data without the assistance of a trustworthy third party, such as a therapy facility. With BC’s support, the framework can withstand unwanted access or a single point of failure. Although the BC only maintains the treatment metadata’s immutable hashes, the actual multimedia data, depending on the application’s needs, audios, videos, photographs, or other augmented reality therapeutic data is saved off-chain in a decentralized database. This functionality allows you to make use of metadata’s immutability while annotating or upgrading multimedia big data [104]. Shynu et al. [120] proposed a secure BC with FC integration healthcare service for illness forecast. When developing projections, cardiovascular disorders are considered. The patient’s health data is initially gathered from fog Nodes and stored on a BC. When compared to existing neural network methods, the suggested approach achieved a prediction accuracy of over 81%.

#### Fraud detection

Fraud detection is the process of checking a document or other data system to see whether there has been any tampering with the data or other harmful activity [164]. The focus here is on how BC can protect FC from attacks. Jeong et al. [223] proposed creating a secure FC system using a reliable distributed BC. IP spoofing, Sybil attacks, and single point of failure may all be prevented with our suggestion. The digital signature utilized in the transaction creation process ensures authenticity and non-repudiation in this proposal. Because it is based on a BC, which is a distributed ledger, it can effectively restore or alternate a downed FC even when it is offline. Stanciu [149] presented a study based on the IEC 61499 standard that uses BC technology as a foundation for hierarchical and distributed control systems. Hyperledger Fabric was chosen as the BC solution, with function blocks being implemented on a supervisor level as smart contracts [149]. Liang et al. [222] suggest utilizing cross-BC-enabled FC to provide safe service detection for the Internet of Multimedia Things (IoMT). An extensible cross-BC design based on FC is provided initially to avoid tampering and espionage during the trust evolution process, in which separate parallel BCs may be coordinated to communicate hidden geographic data and app trusted proof. The smart contract in the BC-based Ethereum is meant to allow Turing complete computing [222].

Misra et al. [147] recommended using a private BC network to implement a Software Defined Networking (SDN) architecture in a fog-enabled IoT ecosystem to prevent such hostile attacks against controllers in real-time. If the miners discover incorrect flow rules, BC permits the SDN devices/fog nodes to revert to a previous flow rule while flagging the accused controller. The authors also recommended encrypting the data before placing it into the blocks, which would help protect the data from unauthorized users [147]. Moreover, Rathore et al. [161] BC technology was offered as part of an SDN-based decentralized security architecture. SDN is in charge of providing an optimal attack detection model by continuously monitoring and analyzing data. The single point of failure concern in the present design is mitigated by BC’s decentralized threat detection [161].

Gul et al. [168] proposed a business model for the healthcare industry that uses BC to link the FC and the cloud. Certain data in the healthcare industry can be analyzed for prediction, and companies can plan before disaster strikes. Many attacks are thwarted since there is no direct contact between the BC layer and the IoT layer. Because the company can predict the course of business and make decisions appropriately, this fusion makes business more productive. Kumar et al. [221] employed two Artificial Intelligence (AI) approaches, random forest and XGBoost, to offer the proposed security framework full autonomy in decision-making skills. An interplanetary file system is recommended for distributed storage and data load balancing. To identify DDoS assaults in smart contracts, the authors presented a distributed system based on FC. The suggested distributed framework’s findings demonstrate that it is extremely successful at identifying numerous assaults in the BIoT network, such as DDoS and other current attacks [221]. Kumar et al. [45] demonstrated how the integration of BC using the PoW consensus mechanism can enhance FC security.

Sharma et al. [73] presented a novel Distributed Mobility Management (DMM) solution based on BC technology for flattened FC. The suggested solution can deal with hierarchical security concerns while maintaining network layout. It uses three BCs to meet the needs of completely distributed security while also resolving the de-registration difficulties that plague previous DMM systems. Furthermore, the distributed BC approach aids in the prevention of DDoS, backward broadcasting attacks, session hijacking, and impersonation attacks. It also encourages the use of de-registration rules. Sivasangari et al. [181] presented a BC with FC integration design to identify security threats at the cloud layer, resulting in a reduction in IoT security attacks. The elliptic curve cryptography-based proxy encryption is used in the proposed design.

#### Confidentiality

Confidentiality refers to the assurances that the data may only be accessed by authorized users or nodes. Other nodes are unable to comprehend the private and secret information that each node possesses [192]. Farhadi et al. [224] explored how distributed BC ledger technology may be utilized to address Confidentiality, integrity, authenticity, non-repudiation, and availability challenges in FC architecture as decentralized computing support [224].

Gao et al. [48] provided a new framework called SGX in the IoT-cloud medical health (IoMT) using BC with FC integration to maintain a trusted environment and data confidentiality. To maintain the highest level of data protection, only a portion of the relevant diagnostic data can be given to the medical facilities in need. Curious data processing facilities, on the other hand, will potentially contribute to data leakage. FC and BC were combined to provide a new platform to address these issues. Mohapatra et al. [229] presented a secure data exchange system for IoT devices based on BC with FC integration. The authors proposed two software agents: a BC creation software agent deployed in FC, and a network of IoT device monitoring software agents. Block addition by an approved IoT device is done with an AES 128-based PoW while hashing in BC was done with SHA 256. To improve FC privacy, Wu et al. [123] integrated BC with FC and leveraged multi-party secure computing technique in smart contracts. Participants can only access the output value of their functions using this technique, which encrypts output and input. Simultaneously, the BC may verify and agree on the findings calculated by this technique across the whole network.

#### Integrity

Data integrity guarantees that the message’s content is not tampered with during transmission [211]. As a result, unlawful data production, deletion, or alteration is prohibited [192]. By allowing all network members to collectively own and validate data, which was previously handled by a centralized server, BC enhances transaction record integrity and dependability. The technology may minimize brokerage fees and construction expenses thanks to distributed data management, while also ensuring high levels of data integrity and security [61]. Kumar et al. [87] argue that the BC maintains data integrity, security, and trust in a decentralized manner. Accordingly, the authors have proposed the BlockEdge framework, which brings these two enabling technologies together to solve some of the existing IIoT networks’ most pressing challenges [87].

A BC-based crowdsensing framework was presented in Gu et al. [127] to deal with security risks, which helps validate the authentication of supplied sensor data and resists record tampering. Guo et al. [227] offered a lightweight encryption system with outsourced decryption. Encryption is the process of converting an original text or data into an alternate version known as ciphertext in order to ensure data confidentiality [32]. Although outsourced decryption reduces the data user’s computing overhead in an attribute-based encryption system, the ciphertext is uncontrollable, and the data owner cannot ensure the data’s accuracy. The proposal guarantees that ciphertext is verifiable, allowing the user to quickly verify for accuracy. Moreover, using BC, the authors enclosed the hash value of the public parameter, the original and modified ciphertext, as well as the transformed key into a block, allowing for tamper-resistance against both internal and external attackers [227].

Jang et al. [193] presented a novel BC with FC integration architecture for IIoT that prevents data falsification by changing existing centralized database methods to distributed types based on BC. They presented a technique to organically manage the IIoT ecosystem by splitting the proposed system structure into cloud, FC, and IoT devices. Users are transferred to the cloud to assure integrity, stability, and scalability. The authors recommended using a fog node to handle smart contracts and transaction verification to improve network latency (the required time for data to move from one location to another) and throughput. For 5G-enabled drone identification and flying mode detection, Gumaei et al. [58] proposed a system that integrates a Deep Recurrent Neural Network (DRNN) with BC. Raw RF signals from various drones in various flight modes are remotely detected and gathered on a cloud server to train a DRNN model, which is subsequently distributed to edge devices for identifying drones and their flight modes. The suggested framework uses BC to ensure data integrity and security [58]. Without a tamper-proof audit, centralized compute offloading poses a security risk. It was unable to protect against false reporting, free-riding, spoofing, and repudiation attacks. As a result, Huang et al. [94] used BC technology to create a decentralized parked vehicle aided FC. Smart contract executions arrange and validate request posting, workload completion, task appraisal, and reward assignment automatically. To reduce security threats, network operations in computation offloading become transparent, verifiable, and traceable [94].

#### Availability

Availability is a critical component of security services, assuring that the system and other apps continue to function in the event of a malfunction or hostile attack [192]. Muthanna et al. [204] proposed an IoT framework that uses a fog node layer managed by an SDN network to deliver high availability and reliability for delay susceptible applications. BC was used to guarantee that decentralization is done safely [204].

Current Agri-Food supply chain provenance and traceability applications are controlled by a centralized technology, which allows the opportunity for unresolved issues and key concerns, such as data integrity, manipulation, and single points of failure [137]. The transaction records are fault-tolerant, immutable, transparent, and fully traceable thanks to BCs [137]. Caro et al. [137] proposed AgriBlockIoT, a completely decentralized, BC-based traceability system for the Agri-Food supply chain that can seamlessly connect IoT devices that produce and consume digital data throughout the chain. They created and deployed such a use-case, establishing traceability using Ethereum and Hyperledger Sawtooth, two distinct BC implementations [137].

#### Insights and discussion

Due to the immutability of the BC, tampering with the data kept in the system is unlikely, and participants’ identities and data integrity may be assured. The data in the BC contains the whole transaction history, which is hashed to keep the ledger secure. As a consequence, BC can ensure that devices are connected (e.g., through smart contract-verified transactions). Fabricating data is almost impossible in the BC system due to the joint monitoring of linked fog nodes (i.e., the attacker will have to alter all of the data on the connected fog nodes, in order to fabricate the data). As a result, BC is protected by distributing data over a large number of linked fog nodes. Authors proposed several architectural designs to support security in FC environment ([81, 84, 104, 120, 139]): to protect against frauds ([73, 147, 149, 164, 168, 181, 221–223]), to enhance and achieve data confidentiality ([48, 123, 192, 229]), to enhance and achieve data integrity ([32, 58, 61, 87, 94, 127, 193, 227]), and to achieve data availability ([137, 192, 204]). The majority of the selected studies under this category reported that BC can help against fraud attacks in FC, followed by data integrity purpose, and the least purpose mentioned was to achieve data availability.

### Privacy

Messages including identity, location, and other personal data are used by many apps and services. As a result, maintaining one’s privacy is critical. The rising demand for FC systems is creating a huge amount of sensitive data. This section discusses the privacy-related purposes including privacy support, identification privacy, data privacy, and location privacy.

#### Privacy support

Several studies have reported that BC can enhance the privacy of FC, in general, as follows. The use of Consortium BC in conjunction with the Transport Layer Security Protocol (TLSP) maintains security and privacy while reducing the requirement for a third party [143]. Pavithran et al. [169] proposed a privacy-preserving BC architecture for IoT. The proposed architecture is well-suited to event-driven IoT devices, and it makes use of the edge and cloudlet computing paradigms, as well as Hierarchical Identity Based Encryption (HIBE) for privacy protection, in which the ciphertext comprises only three group components, and decryption needs only two bilinear map calculations. Uddin et al. [162] suggested a decentralized eHealth architecture based on BC technology. To guarantee patient privacy while outsourcing duties, a patient agent program uses a lightweight BC consensus mechanism and a BC leveraged task-offloading algorithm [162].

Huang et al. [217] developed a fair three-party contract signing mechanism based on BC. To achieve fair trade, the suggested structure employs the verified encrypted signature and the BC. As a result, if a dishonest party aborts after obtaining the present product, it will be punished financially [217]. Gai et al. [93] developed a permissioned BC-edge architecture for smart grid networks to solve two fundamental smart grid concerns: security and privacy. To ensure the legitimacy of users, the authors employed covert channel authorization mechanisms and group signatures [93]. Smart contracts on the BC were used to create an ideal security-aware approach. The efficacy of the proposed technique has been validated for the proposed model [93]. Guan et al. [115] proposed a smart grid scheme for BC-based dual-side privacy-preserving multi-party computing. To assure the security of multi-party computing in edge nodes (e.g., summing), the scheme uses the data segmentation technique. To improve system security and eliminate reliance on trustworthy third parties, the consortium BC and smart contract were used [115].

A decentralized and privacy-preserving charging method for electric cars has been suggested by [88]. The BC system is installed on distributed FC nodes, allowing for a decentralized and secure storage environment. The privacy in the charging process may be maintained by integrating mutual authentication, smart contracts, and BC-based storage [88]. Nadeem et al. [175], in the CRVANETs ecosystem, presented an effective and secure BC scheme-based distributed cloud architecture. Instead of using traditional cloud architecture, on-demand sensing and minimal cost were used to protect the drivers’ privacy. The proposed architecture provides drivers with the necessary security for future autonomous driving [175].

Qu et al. [70] presented the FL-Block system, which allows end devices to communicate local learning updates with a BC-based global learning model that is validated by miners. The FL-Block, which is based on this, allows autonomous machine learning without the need for a central system utilizing a BC PoW consensus technique to manage global coordination [70]. Zhang et al. [116] presented BPAF, a BC-enabled, secure, and privacy-preserving authentication protocol for FC-based IoT devices, which achieves secure fog node authentication without infringing on the privacy of authenticated users during the authentication phase. Hyperlegder Fabric was chosen because it is more scalable and efficient than Bitcoin and Ethereum [116].

#### Identification

Identity privacy guarantees that the identity of a peer or node is hidden from the rest of the network. BC-based identity management integrating access control method was developed by [209]. Self-certified cryptography is used to perform network entity authentication and registration. A Bloom filter-based access control system was also created and linked with identity management. For secure transmission, a lightweight secret key agreement protocol based on a self-authenticated public key was also created. These techniques operate together to offer authentication, auditability, and secrecy for IIoT data [209]. To improve the performance and practicality of FC, Jung et al. [174] suggested a user-friendly FC architecture. According to the recommended design, clients enroll their devices in the fog portal which acts as an intermediary between the resources of each local network and the IoT service [174].

BC with FC integration can solve the problem of identifying, authenticating, and verifying healthcare IoT devices in a decentralized context [172]. Accordingly, Shukla et al. [172] proposed a new solution to the aforementioned dilemma, integrating FC and BC. This solution used the Advanced Signature-based Encryption (ASE) method (a type of digital signature that uses an enhanced certificate to verify the signer) for healthcare IoT device authentication [172]. Tang et al. [136] used a combination of BC and FC to verify each fog server’s identity and create a secure offloading system. A BC-based offloading mechanism was provided to reduce query time and offload security for potential fog servers. A BC-based technique, on the other hand, has inherent limits. All transactions should be recorded to a single copy BC database on each server. If a fog server can handle various queries at once, there will be a large amount of synchronization overhead as a result of this [136].

Wang and Jiang [218] proposed a 2-adic ring identity authentication system that inherits the 2-adic ring’s strong key distribution and great validation efficiency, and this algorithm includes trading node supervision and identity hiding functions by design. The consortium BC was used for this system [218]. Yang et al. [61] looked at how to manage identifiers effectively with BC technology in a named data networking context. By establishing a transaction using the identification’s content name, the suggested system does not reveal a specific user’s identifier. Using an identifier split management approach, the identifier may be safely kept and controlled [61]. Zhu and Badr [129] proposed a hybrid IoT architecture that combines FC with a trustless IoT environment to assure security. Users may easily manage smart devices by establishing tamper-proof digital identities and building a new class of authentication and authorization methods for the IoT by enabling this architecture with BC-based social networks. Fog nodes may also manage all IoT entities’ identities and relationships, as well as implement IoT security measures [129].

#### Data

To protect data privacy, we must ensure that only authorized nodes have access to the data. Data privacy is another important issue for FC [60]. Lautert et al. [146] proposed architecture for tracking data provenance in a distributed FC over a large region. Using software services that maintain the information consistent across all interested parties in the cloud, the architecture presented in this article allows quick and accurate data provenance for clients operating in the FC. The suggested architecture is based on the well-known W3C Prov provenance concept, which makes the framework easier to use. The authors created a client and web services application that allows users to store and exchange provenance information in a BC using open standards [146]. To protect IoT data, Liu et al. [188] presented a decentralized access control mechanism based on BC with FC integration. To encrypt IoT data before uploading to the cloud, this technique employs mixed linear and nonlinear spatiotemporal chaotic models, as well as the least significant bit. The evaluation showed that this mechanism can alleviate the problem of a single point of failure and ensures the privacy of IoT data.

In vehicular fog, there are still several issues with the secure and reliable transmission of sensory data. To address these concerns, Kong et al. [97] proposed a verifiable sensory data collecting and sharing method in vehicular FC using a permissioned BC. By integrating the homomorphic 2-disjunctive normal form cryptosystem with an identity-based signcryption method, the proposed technique achieves the safe and verifiable computation of the average and variance of the collected vehicular sensory data during the data collecting phase. Concurrently, the author used a permissioned BC to maintain a tamper-proof record of the sensory data collected, ensuring reliable and efficient data sharing [97].

#### Location

The third component of FC privacy that should be considered is location privacy. The location of nodes transmitting or receiving data must be known only by authorized nodes [192]. Li et al. [198] suggested a collaborative-ride hailing service that preserves privacy using BC-assisted vehicular FC. It anonymously verifies users and only reveals a targeted user if all collaborating service providers are present, with no need for a trusted authority. The authors used a consortium BC to track c-ride data and build smart contracts to connect passengers and drivers. Location authentication, driver screening, and destination matching are all supported via private proximity tests and query processing. They also tweaked Zerocash to enable anonymous payments and fight against double-spending assaults [198].

Kang et al. [83] developed a privacy-preserving pseudonym system with hierarchical architecture. Pseudonyms are created in real-time and supplied to cars. Safe communication methods for privacy preservation are intended for secure and effective pseudonym management. The authors also demonstrated a situation-aware pseudonym shifting game for automobiles that uses context awareness to alter pseudonyms. The suggested architecture enables safe communication and privacy preservation for cars, according to the security analysis [83].

Patwary et al. [165] suggested a distributed location-based device-to-device mutual authentication system for fog devices at the FC layer, without relying on an intermediate third-party system. Using Ethereum smart contracts, they evaluated BC technology to execute the mutual authentication process. Only a few keys are required by the fog devices for authentication. As a result, the suggested approach satisfied security criteria such as data integrity, confidentiality, mutual authentication, and device anonymity. The suggested technique is computationally efficient, according to the performance evaluation. However, due to the location validation procedures conducted, the suggested system needs greater computing overhead in some situations than previous approaches [165].

#### Insights and discussion

For BC, privacy-preserving strategies based on encryption approaches are evolving, allowing users to become anonymous and have the ability to manage their personal data (e.g., what, whom, and when personal data can be shared in each transaction). Authors proposed several mechanisms to enhance privacy ([70, 88, 115, 116, 162, 169, 217]); to enable and enhance identification ([61, 129, 136, 172, 174, 209, 218]), to ensure data privacy ([97, 146, 188]), and to enhance location privacy ([83, 165, 192, 198]). The majority of the selected studies under this category reported that BC can enhance the level of privacy, in general, followed by identification, and the least purpose mentioned was to achieve data privacy.

### Access control

The tactics or strategies (countermeasures) employed to ensure security goals are referred to as access control [12]. Secure access to data can be ensured using BC in cloud-FC-IoT architecture [186]. This section discusses access control-related purposes including authentication, authorization, and key management.

#### Authentication

Authentication makes sure users are who they say they are. Malicious nodes, fraudulent communications, and unregistered entities are all targets for authentication techniques [211]. Authentication has been identified as a significant problem in FC [14]. Hewa et al. [52] offer a BC with FC integration security service model that runs on FC. Due to the use of BC, the proposed model ensures privacy and authentication. In comparison to current systems, the suggested model demonstrated a higher degree of security and performance. Secure real-time data on items in transit and supply chains necessitates bandwidth with capacity that the present infrastructure cannot provide. To address this challenge Jangirala et al. [121] proposed LBRAPS which is a new lightweight BC-enabled RFID-based authentication mechanism. Only one-way cryptographic hash, bitwise exclusive-or, and bitwise rotation operations are used in LBRAPS [121]. When a regional fog/cloud demands a lot of verification, it causes traffic problems and delays in the master fog/cloud. Kwon et al. [199] proposed a multi-fog/cloud authentication method based on BC to tackle the problem. To overcome this issue, this system distributes an excessive amount of authentication requests around the fog/cloud region. By unifying dispersed multi-fog/cloud throughout the BC network, it increases authentication times [199].

Yao et al. [75], for distributed vehicular fog services, developed a BC-assisted Lightweight Anonymous Authentication (BLA) approach. BLA can benefit from the following: 1) Implementing a flexible cross-data center authentication system in which a vehicle can choose whether or not to be authenticated while entering a new vehicular fog data center. 2) Establishing anonymity and entrusting vehicle users with the task of maintaining their privacy. 3) It is lightweight due to the lack of interaction between cars and service managers, as well as the elimination of communication between SMs during the authentication process, resulting in a considerable reduction in communication delay. BLA provides these benefits by integrating contemporary cryptography and BC technology uniquely [75]. To establish a secure smart vehicle system, Baker et al. [152] presented a lightweight system that uses BC for authentication. To develop the system, the authors used 5G and federated learning in FC. When compared to the present cloud-based framework, the proposed system showed a high enhancement in security level.

#### Authorization

The authorization ensures access to a resource only for authenticated users. Authorization is another important aspect of FC security [2]. The use of the BC idea and the Ciphertext Policy Attribute-Based Encryption (CP-ABE) method, as well as their integration, allows fog nodes in the same fog federation to conduct the authorization process in a distributed way [135]. A user can have several features under the CP-ABE method, and each feature can be shared by several users at the same time [32]. Silva et al. [216] presented a software architecture based on FC to make medical record management simpler. In this design, BC is utilized to allow fog nodes to conduct the authorization procedure in a distributed way. As a result, the traditional authentication architecture’s single point of failure is eliminated, allowing each fog node to function independently and autonomously [216].

To protect data and networks in vehicle FC, Kang et al. [96] incorporated BC into the authorization procedure. This integration improves data sharing and integrity by ensuring data traceability, protecting data security sharing, and mitigating data security concerns associated with centralized data storage through the automated execution of smart contracts [96]. Gai et al. [93] suggested a paradigm that combines BC with edge computing in smart grid networks. Using the BC, this model adds another authorization level. Furthermore, it improves security by utilizing secure communication methods. Also, because the model uses group signatures and the group members don’t know each other, the level of privacy is increased [93]. Khaydaraliev et al. [182] presented a decentralized IoT access control solution. To safeguard device access, the system uses Ethereum Smart Contracts. The evaluation assumes an increase in IoT device access control levels.

#### Key management

Data on the FC must be protected using a variety of cryptographic procedures, which necessitates the use of keys to allow those cryptographic operations; hence, some form of encryption/access control is required [164]. Chen et al. [112] proposed a BC-based key management scheme in FC-based IoT systems to manage secure keys and develop secure group channels. The designated prover PoW (DPPoW), the enhanced PoW mechanism, is used in the proposed main control scheme. This scheme achieves data recovery, conditional anonymity, non-repudiation, conditional anonymity, and resource authentication [112]. Lei et al. [56] presented a system for securely managing keys in a heterogeneous vehicle network. Security managers play a significant role in the system by collecting vehicle departing information, enclosing blocks to transport keys, and then performing rekeying to cars within the same security domain. The framework’s first component is a new network architecture based on a decentralized BC structure. The BC was presented to make distributed key management in diverse vehicle domains more straightforward. In the second section of the framework, the dynamic transactions receivable is employed to reduce key transmission delay during vehicle handover [56].

Wang et al. [122] proposed a BC-based mutual authentication and key agreement protocol for smart grid systems. The protocol may provide efficient key management and conditional anonymity without the use of other complicated cryptographic primitives by utilizing BC [122]. Arun et al. [144] created a method that permits authentication between edge users and freshly added fog servers. The technique instructs the fog servers to keep one secret key per user, with the user performing hash-based encryptions and decryptions. When sensitive data is transmitted between users and nodes, the proposed system uses the BC method to ensure data integrity. All edge and fog nodes are surrounded by a secure system, which records transactions between nodes in blocks that are hashed. Any malicious edge server that enters the network is detected using the ledger kept at the nodes [144]. Tomar and Tripathi [189] suggested a key exchange protocol and mutual authentication mechanism based on BC with FC integration architecture. To ensure message security, the shared key is created between the FC, smart meter, and cloud server. This mechanism’s examination reveals an increase in the amount of access control.

#### Insights and discussion

Malicious activities are detected using BC-based services. When a hostile attacker changes the data in a block, the block’s hash value changes and the block turn to be invalid. By facilitating data access online, the usage of BC, on the other hand, may ease the data analytics lifetime. Certified users can have access to data without having to go through additional checks if various units in a company are involved in a shared BC, for example. Authors provides several mechanisms to ensures and enhance authentication ([52, 75, 121, 130, 171, 183, 199]), to enable and enhance authorization ([93, 96, 135, 182, 216]), and provide key management ([56, 112, 122, 144, 164, 189]). The majority of the selected studies under this category reported that BC can enhance authentication in FC, followed by data key management, and the least purpose mentioned was to enhance authorization.

### Trust management

Trust is defined as the degree to which two nodes accept each other for a certain activity [228]. A technique for building a trust connection between entities is trust management. Trust management is critical, but it is also very energy-intensive, making it unsuitable for resource-constrained devices like those that make up the IoT’s sensing layer [151]. It may be thought of in two ways: as a process of making an entity trustworthy for other entities, and as a process of evaluating other entities’ trustworthiness from the perspective of a given entity [230]. This section discusses the trust management-related purposes including trust support, reliability, transparency, reputation, QoS, and payment management.

#### Trust support

Using BC technology might give several advantages, including a trustworthy workplace [57, 109]. The capacity of the BC system to work successfully in a P2P environment without the involvement of a trusted third party is referred to as trustworthiness or trust-free. It is becoming more possible to transcend the constraints of traditional trust management techniques, thanks to the rise of BC as an immutable ledger technology and the promise of trustless smart Oracles and smart contracts [160].

Cinque et al. [151] demonstrated how to use BC technology to create a federated trust management architecture in which fog/edge nodes help with trust value provisioning and calculation for sensor nodes. Their design has been subjected to a qualitative assessment of the degree of protection it provides. They built a proof-of-concept on top of the Hyperledger3 platform, which is an umbrella project for open-source BCs and related tools [151]. In their work, Kochovski et al. [160] deployed a new trust management technique to handle extremely dynamic and complicated distributed smart application scenarios. This technique counts on the traceability, transparency, and autonomy aspects of BC-based services. By opportunistically combining BC with SDN and container orchestration technologies, Ceccarelli et al. [103] studied how to handle dispersed trust information and allow trusted configuration operations in the IIoT. They concentrated on how the widespread deployment of such technologies may make specialists’ interventions on industrial equipment both easier and more reliable. They proposed the creation of a software architecture to ease the management, setup, and evaluation of IIoT systems for this purpose [103].

Dewanta and Mambo [113] developed a bidding-price-based transaction for vehicular FC service in rural areas to establish mutual trust among vehicles. It is impossible to provide a reliable vehicular FC operation without confidence between vehicles. Therefore, the proposed approach facilitates mutual trust between the client and server vehicles, as well as payout assignment depending on transaction appraisal, without the use of a trustworthy third party to function as a validating agent [113]. Gao et al. [74] looked at how to use a mix of BC and SDN to run Internet of vehicles systems under 5G and FC paradigms. Due to the ubiquitous processing that happens, this proposal helps to ease the burden on the controller by sharing management duties between the BC and the SDN [74].

Jayasinghe et al. [219] proposed TrustChain, a new privacy-preserving BC that combines the capabilities of BCs with trust principles to solve problems with existing BC designs. TrustChain is built in such a way that it only saves information that the users have permitted to store. Techniques such as Zero-Knowledge Proof (ZKP), encryption, and anonymization were used to keep sensitive information hidden while interacting with key stakeholders and assessing trust without compromising privacy [219]. Wu et al. [107] presented the BlockEdge framework, a BC-based framework that allows edge-centric networks to trust collaborative services. BlockEdge uses decentralized accountability and automated incentives to encourage additional distributed edge nodes to function as detectors in verifications. Detectors can earn incentives if they find an untrustworthy outcome, and misbehaving stakeholders can be held liable for damage or accuracy. Furthermore, all stakeholders may benefit from the creation of a trust reputation system, which can serve as an authoritative reference for the selection nodes without relying on a centralized authority [107].

#### Reliability

To ensure the reliability and credibility of source data in FC, Fan et al. [114] suggested a BC-based scheme. This scheme, in particular, assists in ensuring that data is immutable during handling and transmissions, as well as identifying malicious nodes. An attribute-based signature was used to ensure lightweight in this method. This signature makes authentication easier, and BC enables the creation of a secure communication channel that reduces the possibility of data tampering and allows for real-time synchronization [114].

Bonadio et al. [190] proposed an integrated system architecture based on the FC, which was used to establish complete context awareness for the vehicular ad hoc networks and, as a result, to react to traffic anomalies. Hu et al. [156] proposed an organic agriculture supply chain-style trust architecture that has a significantly superior cost-to-efficiency ratio. Furthermore, they split all stakeholders into four roles based on this style scenarios, providing a unique consensus technique to control information flow [156]. Xu et al. [220], in-network computing situations, proposed a unique BC-based technique for shielding clients from doubtful services. The BC was created to keep track of all the legitimate states of edge service providers and off-chain IoT services, allowing them to eliminate untrustworthy or rejected services Via supplier authentication and service validation [220].

The digital cryptocurrency GlucoCoin was used to build an incentive scheme to encourage patients to contribute fresh data [206]. A BC is used in such a system to perform smart contracts, such as automating sensor purchases or rewarding users who contribute to the system by contributing their data. The suggested system enables the crowdsourcing of patient data as well as the creation of unique mobile health (mHealth) apps for monitoring, diagnosing, analyzing, and implementing public health activities that can help in disease management [206].

#### Transparency

Lautert et al. [146] proposed architecture for tracking data provenance in a distributed FC over a large region. Localized fog nodes have control over what is made public on the cloud, whereas BCs give transparency. Mondal et al. [80] suggested an IoT architecture inspired by BC for establishing a food supply chain that is transparent. The design employs a proof-of-object-based authentication system, similar to the PoW technique. At the physical layer, a Radio Frequency Identification (RFID)-based sensor was integrated, and at the cyber layer, BC was used to complete the architecture. The RFID gives the product a unique identification as well as sensor data, which helps with real-time quality control. At each location, the BC architecture assists in the creation of a tamper-proof digital database of food products [80]. The use of BC technology may improve transparency, information flow, and managerial capacity, allowing farmers to connect more effectively with other parts of the supply chain, particularly consumers [184].

Utility providers’ interactions with their consumers over power usage have improved since the advent of smart grid technologies. However, because the readings are done through the Internet, there is a risk that the data will be compromised if it falls into the wrong hands. Furthermore, because they are not privy to the data, most consumers have no idea why they are paying such high prices or which gadgets use the most power. Accordingly, Gao et al. [62] developed the sovereign BC technology, which offers transparency and provenance to address the issues described above. A smart contract was also created, which executes pre-defined operations to establish a trust-based platform between network members. This platform allows the user to monitor how the power is utilized and it also gives a platform where neither side can manipulate the situation [62]. Ngabo et al. [202] suggested a BC-based security system that uses an elliptic curve cryptography digital signature to enable a decentralized ledger database, providing transactional transparency, immutable safety, and preventing tampering with health records at the FC layer.

#### Reputation

A reputation is an opinion about another thing held by an object (human or machine) [228]. Almost all technical and non-technical systems rely heavily on the reputation of trust management [63]. Debe et al. [72] proposed a decentralized trust model to preserve the reputation of publicly available fog nodes. Users’ views regarding their previous encounters with public fog nodes are taken into account while maintaining the reputation. The suggested model is constructed using public Ethereum BC and smart contracts technologies to allow distributed trustworthy service provisioning between public fog nodes and IoT devices [72]. Iqbal et al. [95] proposed a safe FC paradigm in which roadside units transfer duties to adjacent fog vehicles based on reputation scores kept on a distributed BC ledger. Accordingly, the decision model can choose from a pool of trustworthy cars for any incoming jobs [95].

Sun et al. [170] proposed a reputation-based crowdsourcing BC framework. A user, FC, and cloud make up the three-layer chain architecture. This architecture paired with the Hyperledger Fabric consortium BC network can provide privacy protection (i.e., the channel mechanism ensures transaction anonymity - members outside the system cannot see all details on the channel, including purchases, members, and channel content). This architecture can also provide reputation management. The legal identity is a representation of the entity’s past conduct. Its reputation status is revised regularly based on recent activity. Adversarial can be avoided by lowering the trust status of all service members who engage in untruthful behavior [170].

#### Quality of service

Understanding BC with FC integration is essential for enhancing cyber-physical systems in terms of Quality of Service (QoS) (a definition or estimation of a service’s overall performance [64, 153]. Because of the IoT’s rapid growth, ensuring QoS over FC networks may be difficult. QoS measurement approaches have traditionally relied on a centralized organization that gathers data and analyzes service performance with the help of specialized agents [63]. Traditional approaches, on the other hand, are incapable of coping with a diverse and distributed set of services like the IoT. We must be able to gather, retrieve, and update proper quality data regularly to manage QoS in distributed services [156]. The BC participant approach guarantees the data necessary to assess the quality of IoT services is reliable. To offer great QoS in highly mobile networks, secure and trustworthy transmission is essential [156].

To solve difficulties related to QoS and data storage, Bouachir et al. [64] suggested industrial cyber-physical systems based on BC with FC integration. Distributed data storage and management over the FC, according to the author, are potential answers to data storage and QoS issues [64]. As an approach to eHealth services, Islam et al. [159] suggested a novel BC with FC integration management system focused on the creation of clustered-based extracted features for the detection of human activities. Bag-of-features, based on Speed-Up Robust Features (SURF), were utilized in the proposed system to pick interest spots for human actions in films. The suggested system’s efficiency and accuracy are improved by using the Error-Correction-Output-Codes (ECOC) method, which allows for classifying multi-class actions [159].

To build confidence in smart apps and the underlying decentralized system, Kochovski et al. [160] looked at several factors that must be evaluated and applied. While certain trust characteristics can be gained through expensive on-BC activities, others can be achieved using less expensive off-BC techniques, such as the usage of data QoS monitoring. To attain good QoS of smart apps, the authors use off-BC QoS monitoring data acquired via a trustless Smart Oracle, as well as a Markov decision-making mechanism that rates the various FC/cloud node providers to pick the best fog node for the AI component of the application’s deployment [160]. Debe et al. [76] proposed a new system for monetizing BC-based services and automating bitcoin payment for services delivered by fog nodes. The suggested method is trustworthy, decentralized, and automated, which enhances QoS and customer satisfaction. The suggested approach governs interactions between FC and devices using the Ethereum BC and its inherent smart contract capabilities [76].

#### Payment

The incentive and penalty systems utilized by the fog node for BC’s participants are referred to as payment, in this context. Debe et al. [91] proposed a decentralized reverse-bidding method based on BC and smart contracts’ main characteristics. They created a system that allows devices to start the bidding process by requesting services from nearby fog nodes that respond with bid proposals. The suggested method guarantees that all fog nodes on the network compete for the bid fairly and equitably. The automatic payments after the service are included in the bidding procedure. Ethereum smart contracts were used to implement this solution. This method also included a fog node’s reputation system, as well as a penalty for nodes that misbehave [91]. Moreover, Liu et al. [100] proposed distributed BC-inspired energy coins and data coins.

By utilizing the advantages of smart contracts of BC, Jain and Kumar [213] created a fair and trustworthy incentive mechanism that promotes sellers and buyers to transact. Various economic attributes, such as budget balance, personal reasoning, and honesty, are satisfied by this mechanism. The incorporation of the BC and FC precludes the manipulation of trade-related data. The suggested technique was shown to be effective in identifying the winner and pricing model. Shukla et al. [196] demonstrated a BC-based smart energy trading algorithm and a BC with FC integration-based system for P2P energy trading. The proposed algorithm creates a completely trustworthy, low-latency communication network that allows prosumers to trade energy inside their neighborhood, based on the evaluation results. Boualouache et al. [51] developed a monetary reward strategy for 5G-enabled FC-based vehicle location privacy preservation. This solution makes use of a consortium BC in the FC layer as well as smart contracts to assure pseudonym changing procedures and lower vehicle monetary expenses. This scheme provides appropriate monetary cost management and private verification of blocks, according to the evaluations.

#### Insights and discussion

Because each node in the consortium BC, for example, has access to the data and business norms, the BC’s transaction may be trusted. The BC ledger can now be used to register and exchange nearly anything without the need for a single authority. As a result, a trustworthy and successful network can be initiated. Moreover, by assuring that a fog node is in command of its identification, the immutability of BC gives the necessary reliability and confidence for corporations among nodes. The basic idea is to provide fog nodes identifications that can be verified with BC throughout their entire cycle. A record or timeline is created by a system with an identification, which is managed by a BC. The vast bulk of BCs is open-source, meaning that nodes can see and use their transactions. Users may look up the record of all transactions in the case of Bitcoin thanks to BC transparency. As a result, there will be more openness, which will improve productivity. Bitcoin, for example, is changed when a large majority of network users agree that there is a need for updated code that sounds beneficial. Authors proposed several strategies to ensure trust support ([57, 74, 103, 107, 109, 113, 151, 160, 219]), to enable reliability ([114, 156, 190, 206, 220]), to enable transparency ([62, 80, 146, 184, 202]), to increase reputation ([63, 72, 95, 170]), enhance QoS ([63, 64, 76, 153, 156, 159, 160]), and secure payment ([51, 91, 100, 196, 213]). The majority of the selected studies under this category reported that BC can enhance trust level, in general, in FC, followed by QoS purpose, and the least purpose mentioned was to achieve a high reputation.

### Data management

Another issue of FC is the data management due to the heterogeneity and distributed nature of IoT devices in the FC environment [14]. This section explains how BC with FC integration may help to solve several data management problems, focusing on data management-related purposes including storage, sharing, and validation.

#### Storage

The transitory fog storage is capable of briefly storing data acquired from IoT devices, allowing IoT devices to save frequently requested data and accomplish fast data updates [19]. Data identification, aggregation, and integrity should be used to meet privacy and security needs for data storage [41]. Cech et al. [43] deployed an FC node with BC capability to solve the challenge of storing and securely exchanging sensor data. The authors used the MultiChain BC framework to connect it to the virtualized modular FC gateway. Two new protocols for data storage and access control were built and thoroughly explored in a fog node. The first allows data to be shared with chosen organizations over a public BC channel. The second allows the BC to store streaming real-time sensor data. So, this system enables making non-sensitive material freely available while restricting access to the sensitive sections [43]. Ren et al. [178] developed a technique combining BC and regeneration coding to increase the security and dependability of stored data. Hybrid storage architecture and model were developed. A global BC in the cloud service layer was then created, taking full advantage of the benefits of edge network devices and cloud storage servers. The regeneration coding was used to increase the data storage reliability even further. Furthermore, the local BC was created on IoT terminals, allowing for the second verification. After the data is saved in the cloud, it can be compared and validated against the data in the local BC, enhancing data security even further [178].

By combining FC and the BC, Chen et al. [163] created a three-tier architecture-based data aggregation system that provides significant support for accomplishing efficient and safe data gathering in smart grids. They used Paillier encryption, batch aggregation signatures, and anonymous authentication to create a safe and anonymous data aggregation technique with little computing overhead [163]. El Kafhali et al. [225] presented a distributed BC cloud architecture to efficiently manage the raw data streams generated by the massive number of IoT devices. The suggested design takes advantage of BC, FC, SDN, and Network Functions Virtualization (NFV) techniques. The suggested architecture may greatly minimize the communication time between IoT devices, resource distribution, and traffic loading in the network, making it easier to deploy IoT services [225]. Nkenyereye et al. [208], for 5G enabled vehicle edge computing, suggested a safe and BC-based Event-Driven Message (EDM) protocol. They utilized a lightweight multi-receiver signcryption system that does not need pairing and provides high privacy and security levels, and low-latency operations. EDM records must be stored in a distributed system that ensures EDM’s dependability and auditability. To do this, they deployed a private BC to store EDM records depending on the edge nodes [208].

#### Sharing

Data sharing has to do with determining who should receive and what sort of broadcasting content should be disseminated to protect data [96]. In heterogeneous systems, data sharing is an inherent problem. BCs, as a strong tool for addressing security concerns, may use consensus methods to assure the trustworthiness and irreversibility of computational data [96]. Abdellatif et al. [110] proposed a Medical-Edge-BC (MEdge-Chain) framework for dealing with vast volumes of medical records. The proposed framework, in particular, outlines a healthcare infrastructure that seeks to bring together disparate government institutions into a single national healthcare system by allowing for the rapid and secure sharing and storing of medical data [110].

Storing data directly on the BC results in a huge increase in size. Because previous transactions cannot easily be removed from a BC’s history, rising storage needs would soon transform a fog node acting as a peer of the BC into a substantial cost issue, preventing nodes with low resources from participating [43]. As a result, keeping simply the hash value of the data in the BC can give the same assurances while using far less storage. A calculated hash value has a fixed length regardless of the amount of data. The real data can then be saved differently [43]. On retrieval, the data’s integrity may be checked by recalculating its hash value and comparing it to the one that is stored immutably on the BC [43]. Bai et al. [111] proposed a Multiedgechain structure, from the aspect of real-time operation and stability, that supports a big amount of data and improves on-chain data efficiency to provide cross-chain data sharing for diverse BC platforms. Furthermore, a two-stage Stackelberg game tactic was presented, taking into account the risk considerations and user preferences, to maximize the profitability of computing resource scheduling on the Internet of energy [111]. Ismail et al. [145] proposed a framework to enhance data sharing by employing BC methods and data operations to prevent data from altering. IoT may be used to remotely monitor a patient’s status, as well as follow up and provide information to the appropriate authorities, alerting them to potentially harmful circumstances. The data is obtained from the patient, processed in operations, and then saved to communicate trustworthy and reliable information between the caregivers and the patient [145].

Several research initiatives have recently been completed to allow the collaborative platform to create successful collaboration with the manufacturing, design, and consumer perspectives. However, establishing trust and effectively utilizing consumer perspectives remains a difficulty. As a result, Barenji et al. [167] suggested a BC-enabled FC-based collaborative platform to foster triple communication and collaboration in a secure environment across the manufacturing, design, and client sections. Machine learning was utilized to cluster and categorize customer views in the proposed platform, and FC-based integration across subsystems using BC technology is proposed to increase data integrity and security [167]. According to Shahbazi and Byun [212], BC can shift the smart manufacturing on edge computing servers from a cloud-centric to a distributed system FC architecture. In their proposal, the BC technology makes use of data transfer and production system transactions, while the machine learning method allows for enhanced data analysis of a large manufacturing dataset [212]. Rivera et al. [90] proposed a BC framework to offer a trusted cooperation mechanism between edge servers. A permissioned BC approach is being studied in particular to support a trusted design that also offers incentives for collaboration [90].

To accomplish safe data storage and sharing in vehicle edge networks, Kong et al. [96] developed a reputation mechanism to ensure that cars provide high-quality data. For accurately managing vehicle reputation, a three-weight subjective logic model was used [96]. Yang et al. [157] proposed a smart-toy-edge-computing-oriented data sharing model utilizing HLF v1.0. They set out to address the problem of automatically preserving a trustworthy, tamper-resistant, and distributed ledger by developing smart contracts in a world where people distrust each other. This prototype can streamline the process, save time and money, and ensure that disputes are resolved fairly. This technique also makes P2P data sharing between distant smart toys and other IoT devices easier to install [157].

#### Validation

The data transferred from the fog to the cloud will be altered. As a result, the user of an IoT device will never be able to check the accuracy or integrity of data saved in the cloud [14]. When BC is used in conjunction with FC, data validation guarantees that the access token and digital signature (for example, in the smart contract) are valid before the review is stored [138]. Simpson and Quist-Aphetsi [142] suggested a framework that makes it simple to ensure that a patient’s medical information is accessible across multiple healthcare institutions. The usage of a BC ledger allows databases to utilize timestamps to validate and maintain current patient health information in a centralized data cloud [142].

Tian et al. [138] presented a custom-built public auditing technique for data storage that fulfills security and performance requirements. During the proof generation stage, they designed a tag-transforming mechanism based on the bilinear mapping technique to translate tags generated by mobile sinks to tags created by fog nodes. This technology not only efficiently preserves identity anonymity but also saves time and money throughout the validation step [138]. Li et al. [65] suggested a carpooling method that supports conditional privacy, destination matching, one-to-many matching, and data auditability utilizing BC with FC integration-based vehicular networks. This method verifies users in a conditionally anonymous manner. Also, it uses one-to-many proximity pairing using a private proximity test and extends it to provide a secret communication key between a client and a driver. A private BC was created to keep track of carpooling records [65].

#### Insights and discussion

BC can guarantee safe data sharing because due to its distributed and immutable capabilities. Financial firms can watch each transaction in live time thanks to the data stored in BC, enabling them to examine possibly fraud cases. Hence, the BC with FC integration can assist financial firms in preventing fraud and safeguarding their consumers. Additionally, this integration enables service providers to exchange data with other stakeholders while minimizing the risk of data loss. Furthermore, if the data comes from a variety of sources, the need for repeated data analysis may be avoided because each transaction is recorded in the BC. Smart Contracts can be used to govern the data sharing and storage process in BC. On the other hand, to enable large data communications, BC can assure big data training and avoid data breaches. Authors have introduced several strategies to support data management: to secure data storage ([43, 163, 178, 208, 225]), ensure data sharing ([43, 96, 110, 111, 145, 157, 167, 212]), and to ensure data validity ([65, 138, 142]). The majority of the selected studies under this category reported that BC can enhance data sharing in FC, followed by data storage purpose, and the least purpose mentioned was to achieve data validity.

### Scalability management

If FC is used in conjunction with BC, it poses scalability issues. The transaction efficiency in the BC, which includes throughput and confirmation delay as important parameters, is far too low for FC [98]. This section explores the solutions that have been provided to overcome this issue. In particular, it discusses several BC-based solutions to reduce the scalability issues linked with implementing PoW-based BC in FC. As a result, the suggested solutions rely on PoW with scalability augmentation techniques, as plasma and SDN approaches. However, these are limited in terms of scalability and have significant power requirements. Other solutions used different consensus mechanisms while sacrificing security, privacy, or decentralization. This section, specifically, discusses the scalability management-related purposes including scalability support, regulations, and mobility.

#### Scalability support

Baouya et al. [140] proposed a BC-based architecture for IoT device control that is scalable. Smart contracts were created to make the ledger updating process easier. The suggested architecture is capable of delivering trust on-demand modifications with minimal impact on IoT resources, according to experimental results [140]. Chen et al. [106] designed a secure distributed data management platform for FC in large-scale IoT applications. This addresses one of the key issues: how to integrate data security and storage management for FC in large-scale IoT applications while also enhancing rational interoperability for networked objects [106]. Ziegler et al. [126] proposed a novel system architecture that integrates BC technology with FC using the Plasma framework to address the performance drawbacks. The Plasma framework has the benefit of allowing for a scalable hierarchical design based on sidechains as well as an off-chain scaling method that is independent of the root chain architecture. Plasma has already demonstrated its great potential and distinguishes itself from other off-chaining solutions, owing to its low requirements for the parent BC and ease of implementation. It allows for increased efficiency, which is required for real-world operations [126].

Sharma et al. [77] proposed a model that integrates FC, BC, and SDN. The fog node uses the BC technique to bind all of the SDN controllers in a distributed manner. The performance assessment revealed that, as opposed to conventional core-based cloud computing technology, this model is a more effective approach for offloading data to the cloud and adheres to the necessary architecture principles with reduced overhead. This would provide IoT network participants with low-cost, stable, scalable, and access to the most competitive computer resources on demand [77]. Lei et al. [98] introduced the Groupchain (PoW and PBFT), a scalable public BC with a two-chain structure that is appropriate for IoT services computing FC. The leader group is used by the Groupchain to commit blocks collectively for greater transaction efficiency, and bonuses and deposits are included in the incentive mechanism to oversee the actions of members in the leader group [98]. Lakhan et al. [53] created the BC enables task scheduling algorithm framework to decrease the cost of application’s security and processing. In comparison to current methods, the processing cost was reduced and the security level was enhanced, according to the evaluation.

#### Regulations

The ability to offer a high degree of information security via BC (e.g., smart contracts and Etherium) allows for the creation of a dependable and transparent system of regulation for all transactions [141]. Pan et al. [102] proposed an EdgeChain framework, a new edge-IoT architecture based on BC and smart contracts. EdgeChain includes a permissioned BC that connects edge cloud resources to each IoT device’s account, resource use, and, as a result, behavior. To regulate the IoT devices’ resources that may be received from the edge server, EdgeChain employs a credit-based resource management mechanism. Smart contracts are used to control the behavior of IoT devices and enforce regulations [102]. Stanciu [149] presented a study based on the IEC 61499 standard that uses BC technology as a foundation for hierarchical and distributed control systems. Hyperledger Fabric was chosen, with function blocks being implemented on a supervisor level as smart contracts. The integration with the executive nodes, which are responsible for real process management and based on a micro-services design in which the Kubernetes platform was used to organize container execution across edge resources utilizing Docker containers and the Kubernetes platform [149].

#### Mobility

In many businesses, mobility is becoming increasingly important. The capacity to transfer data is becoming increasingly crucial as smart gadgets, sensors, and other internet-connected devices grow more common [164]. Some solutions, such as a pervasive social network system and a BC-based healthcare data gateway, have been suggested in healthcare to address the issue of mobility and wireless sensing. Lakhan et al. [117] deployed BC for scheduling and offloading of mobility-aware vehicular FC-cloud architecture. The study aims to reduce application connectivity and computing costs while keeping mobility, security, deadlines, and resource constraints in mind. The study proposed a Mobility Aware BC-Enabled offloading scheme (MABOS) to ensure mobility protection. It uses proof of creditability (PoC), PoW, and fault-tolerant techniques to allow multi-side offloading (e.g., offline and online offloading) on the BC [117]. Moreover, multiple access mobile edge computing looks to be an advantageous approach to solve the PoW problems for mobile users in future mobile IoT systems, facilitating BC applications in future mobile IoT systems. Accordingly, Xiong et al. [108] introduced the idea of edge computing for mobile BC. They proposed a cost-effective way to manage edge computing resources [108]. Alotaibi et al. [124] presented fog-based internet-of-smart vehicles combining BC and SDN (SaFIoV) to handle secure communication and load-balancing issues. Utilizing reinforcement learning approaches, SaFIoV properly allocates tasks in the vehicles-to-fog and fog-to-fog layers. The use of BC ensures the security of communication.

#### Insights and discussion

Despite the scalability issue of FC, several authors suggest using BC can decrease the impact of this issue. Several designs proposed for this reason ([106, 140]), using the Plasma framework [126], using SDN capabilities [77], deploying the Groupchain [98], and deploying a scheduling algorithm [53]. Moreover, regulations represent another scalability issue, and hence, the authors suggested using Smart contracts [141], a credit-based resource management mechanism [102], and hierarchical distributed control systems [149] to govern BC with FC integration. On the other hand, mobility in several applications represents another scalability issue for FC. Authors suggest mobility-aware offloading and scheduling systems [117], the idea of edge computing for mobile BC [108], and SaFIoV [124], to manage the mobility of BC with FC integration-based applications.

### Performance

Another issue of FC is the low performance [4]. This section explores the solutions that have been provided to overcome this issue. This section, specifically, discusses the performance-related purposes including resources, latency, energy consumption, and fault tolerance.

#### Resources

Because of the variability of resources required to support a variety of IoT applications, resource management is critical to improving the performance of FC [41]. Hamdi et al. [154] argue that the procedure of selecting an acceptable target fog node for available resources of parked and moving cars should be similar to that of forming a service level agreement in order to ensure that the suitable target fog node is chosen. The various requirements that must be addressed in order to build such an SLA were discussed. Gao et al. [118] proposed BC-Enabled Resource Sharing and Transactions (B-ReST), a novel architecture for resource sharing and transactions in FC networks. Baniata et al. [166] used the ACO algorithm in the FC-BC called Privacy-aware fog-enhanced BC-assisted Task (PF-BTS) model. ACO assists miners in generating solutions through a series of iterations in this model. This guarantees high task assignment security and performance, resulting in reduced computing power and time [166]. He et al. [66] designed a smart contract inside a private BC network that uses Asynchronous Advantage Actor-Critic (A3C), a state-of-the-art machine learning algorithm developed by GoogleMind, to distribute edge computing capabilities, demonstrating how AI can be coupled with BCs [66]. Yang et al. [67] developed a distributed matching mechanism within the context of matching theory to optimize the social wellbeing of fog nodes while assuring that certain fog node mining criteria are met.

Liao et al. [89] proposed a new task offloading mechanism to fix issues such as reducing queuing delay, task offloading delay, and handover expense of missing data while maintaining privacy, fairness, and protection. The authors suggested a QUeuing-delay aware, handOver-cost aware, and Trustfulness Aware UCB (QUOTA-UCB) algorithm based on a subjective logic-driven trustfulness evaluation process. The Merkle hash tree and smart contract were used to achieve “proof-of-computing” and to protect against “double-claim” attacks, “free-ride” attacks, and repudiation attacks [89]. Rahman et al. [92] suggested a BC-based infrastructure to provide secure and private Spatio-temporal smart contracts services for mega smart cities long-term with IoT-enabled economy sharing. Cognitive fog nodes are used to analyze and store geo-tagged multimedia transactions that have been offloaded. It uses AI to gather and analyze important event data, give semantic digital analytics, and preserve results in BC and distributed cloud storage to enable sharing economy apps. The model outlines a long-term reward system that might aid in the security of smart city services such as sharing economies and cyber-physical interactions with IoT and BC [92].

The size of BC data is always growing. To mitigate this issue, Wang [69] proposed the Dewblock, a new type of BC system. A BC client does not need to maintain BC data on this system, yet it also has all of the characteristics of a BC complete node. Dewblock introduces a novel technique in which a client’s data size is decreased but the properties of a complete node are preserved. The essential point is that with Dewblock, the two concepts of BC client and BC node are no longer interchangeable. While a client is lightweight and may be run on a home computer or a mobile device, it collaborates with a distant cloud server to perform the functions of a complete node [69]. Holste et al. [131] demonstrated VarOps, a framework that allows application developers to focus on features that can be reused across various frameworks, resulting in significant productivity improvements while also decreasing administration and maintenance complexity. This was accomplished through the use of automated multi-party smart contracts that could be used for several business models. This increases the possibility of delivering a secure ecosystem of computational resources including data, software components, component repositories, IoT devices, computational infrastructures, and networks [131].

Kong et al. [180] proposed a BC-based resource management system for vehicular FC to increase the security and fairness of resource transactions. They originally introduced the Resource Coin (RC) idea and developed a BC-based secure computing resource trade mechanism based on RC. The roadside unit participates in the BC network as a node, confirming the authenticity of transactions and creating new blocks. Then, using contract theory, they suggested a resource management strategy that would encourage parked cars to donate computing resources so that could perform PoW faster, increase block creation success rates, and earn RC rewards. By providing computational resources, vehicles may get comparable RC prizes. This compensation can be exchanged for car networking resources (communication bandwidth, storage space, and so on) to improve the QoS of the company’s vehicle services [180].

Liu et al. [99] presented a unique BC-based architecture for mobile edge computing video streaming with adjustable block size. The authors devised an incentive system to encourage content providers, video transcoders, and consumers to collaborate. Then, for BC-based video streaming, they introduced a block size adaption technique. To tackle the problem in a distributed manner, they used a low-complexity Alternating Direction Method of Multipliers (ADMM)-based algorithm. The entire problem may be split down into local optimization challenges at each SBS using the ADMM algorithm. The computing complexity can be considerably decreased in this manner [99]. Chang et al. [59] recommended that the BC can be of high help in drone networks. Drones that are used to provide services can act as BC miners, obtaining computational resources as required from each other or an edge computing node, according to the proposed BC-empowered drone networks (BeDrone) [59].

Samaniego et al. [125] developed a solution to the challenge of hosting a BC on standard IoT hardware. The authors looked at how fog and cloud computing architectures may be used in BC-IoT applications. The fog system beats cloud-based systems in terms of latency reaction time under heavy transmission loads, according to the system’s empirical performance evaluation [125]. Savi et al. [134] proposed an architecture in which several FC platforms, each of which manages a dispersed structure if resources from a different administrative domain are needed, can effortlessly merge their capabilities through a BC-based brokerage platform. Tuli et al. [158] proposed the FogBus framework, which can connect various IoT-enabled equipment to FC and cloud infrastructures. The framework makes it easier to deploy IoT applications, monitor resources, and manage them.

Wang et al. [205] looked at the resource contribution mechanism between the fog node and the cloud or users. The authors suggested an approach that uses the BC’s incentive and punishment system to encourage fog nodes to actively contribute resources. The behavior of the fog node in terms of contributing resources, as well as the task completion degree for contributing resources, are packed into blocks and kept in the BC system to create a transparent, open, and tamper-proof service assessment index. The differential game technique was used to describe and solve the aforementioned process, as well as to handle the relationship between the fog node’s optimal resource contribution strategy and the optimal benefit under that strategy [205]. Using a game-theoretic approach, Xiong et al. [78] investigated the interaction between cloud/FC providers and miners in a BC network. They suggested a lightweight PoW-based BC architecture in which the consensus process’s computation-intensive portion is delegated to the cloud or FC. They deployed a two-stage Stackelberg game, in which the profit of the cloud/FC provider and the individual miners’ utilities are jointly maximized. The cloud/FC provider determines the price of the supplied computing resource in the first level of the game. The miners decide on the quantity of service to acquire in the second step [78]. Jiao et al. [79] focused on cloud/FC service provider-miner trade and offer an auction-based market model for optimal computing resource allocation. They developed an approximation method that ensures the accuracy, individual rationality, and computational efficiency of the data [79]. Luong et al. [101] presented the construction of an optimum auction for resource distribution in FC using deep learning. The suggested optimum auction was created with BC applications in mind. The authors demonstrated how to use deep learning to build the optimum auction for the fog resource allocation in the BC network [101]. A BC with FC integration resource allocation and task offloading algorithm was presented in [215] to improve FC performance by concurrently optimizing task resource allocation and offloading decisions.

#### Latency

Cloud computing lowers the cost and resource consumption of smart surveillance systems but at the risk of adding extra delay through centralized systems located far away. It’s a challenge to keep data secure in the heterogeneous cloud-FC-IoT network environment. Mayer et al. [54] introduced the FogChain architectural concept, which includes FC, BC, and IoT. When compared to cloud-like BC architecture, FogChain could achieve a 62.6% quicker response time. Whaiduzzaman et al. [55] created a safe BC method for a FC-IoT architecture. They implemented a BC to address the network performance issues [55]. Gharbi et al. [186] presented a new cloud-IoT distributed infrastructure that supports real-time data transmission, stability, and low latency. It was built on the foundations of three new technologies: FC, multi-agent systems, and BC. Since it is close to IoT computers, FC will significantly minimize the latency. The Multi-Agents System allows for distributed execution and has very effective proactive and reactive capabilities that are very useful in IoT applications. BC technology ensures data confidentiality and allows secure low-latency access to massive volumes of data [186]. To solve the real-time data processing problems of IoT, a SoftEdgeNet model was developed by Sharma et al. [105]. For a sustainable Ff network, the SoftEdgeNet model deploys a novel SDN-based dispersed layered architecture with a BC technique at the fog layer. This model can offer real-time analytics and prevent security attacks. The SoftEdgeNet model not only filters unreliable, fake data early and mitigates external attacker attacks, but it also provides fault tolerance capabilities [105].

Saputro and Sari [150] proposed Lightweight Multi-Fog (LMF) BC, which incorporates FogBus algorithms, and the lightweight scalable BC which uses distributed time-based consensus algorithm. This model was suggested to minimize FogBus latency and enhance availability and integrity. The broadcast domain separation model was used by LMF to enhance integrity. Broadcast domains separate transactions and procedures on a per-broadcast-domain basis. LMF additionally features a location verification system to ensure that requestor transactions are performed in the nearest broadcast domain, as well as to protect brokers from illegal transactions. LMF can improve availability by processing transactions on the closest broadcast domain, reducing latency, and providing a fault-tolerance system that combines lightweight scalable BC and FogBus, as well as a cloud backup method. As a result, when a broadcast domain is attacked, it does not affect transactions and processes in other broadcast domains [150].

#### Energy consumption

BC creation consumes a lot of processing power, which can quickly deplete the computing capacity of fog nodes. Accordingly, Wu and Ansari [68] recommended that the fog ecosystem be divided into fog node clusters, with each cluster’s fog nodes maintaining the same access control list, which is protected by a BC. They modified the BC for the fog node cluster to decrease the amount of computational power and storage space necessary. They also proposed a heuristic approach, referred to as Time aWare computing sEt Allocation algoRithm (WEAR), that uses all available devices to minimize the time it takes to collect block hash values [68]. Singh et al. [203] demonstrated a safe BC architecture and a fog-based architecture network for IoT applications in smart cities. Encryption, authentication, and BC are all used in the proposed architecture to protect sensitive data. The suggested architecture’s objective is to use BC technology to minimize latency and energy consumption while also improving security [203].

In response to commercial data and analytics, real-time apps, and worries about energy saving, Memon et al. [71] proposed a DualFog-IoT architecture that divides the computational resources of the fog layer into two parts: FC/cloud cluster and fog mining cluster. The proposed architecture supports three application request types: delay tolerant, non-real-time, and real-time. For these arrangements, the access point between the device layer and the DualFog layer acts as a filter; real-real-time requests are passed to the FC/cloud cluster, while non-real-time requests are forwarded to the cloud datacenter. Incoming delay tolerant, on the other hand, are held on hold over AP until they reach the size of a block. Once the block has been created, it is sent to the fog mining cluster to be mined [71].

#### Fault tolerance

Fog, like any other paradigm, may not be built in such a way that it is safe and immune to all attacks. Due to obsolete software, vulnerabilities, misconfigurations, and other flaws, malicious adversaries may be able to deactivate or seize control of some of the fog nodes, if not the entire infrastructure [41]. Using game theory, Casado-Vara et al. [226] presented a distributed and self-organized cooperative algorithm. The program was used to analyze data collected by IoT devices. In addition, to increase data security, a BC-based architecture was proposed. This algorithm is performed to enhance data quality and false data detection is a unique feature [226]. Based on an Ethereum BC implementation, a novel architecture dubbed Heterogeneous, Interoperable, and DistRibuted Architecture (HIDRA) was proposed, aiming at resource orchestration in FC-IoT applications [119]. HIDRA is a fault-tolerant, secure, and auditable distributed architecture.

Lallas et al. [132] developed a decentralized IoT-FC-cloud architecture for real-time failure prediction and machine monitoring, in which computationally intensive activities are spread among fog nodes and decision fusion rules are established and managed by the cloud. Whereas, a P2P BC ledger is projected to integrate this architecture and other entities of the physical world, resulting in a more efficient and intelligent supply chain network [132]. Mounnan et al. [148] suggested a new architectural model that uses BC technology to provide access control in the IoT using FC. The proposed solution takes a fresh perspective on a variety of problems. By implementing the policy through smart contracts on the BC network, this solution assures the performance of the identity and authentication procedure. As a result, if the user’s qualities match the policy, access is allowed. Furthermore, because load balancing is implemented using the Min-Min algorithm, this proposal provides greater availability and fault tolerance in fog nodes [148].

#### Insights and discussion

Financial firms may settle cross-border transactions, especially those involving huge sums, in near real-time thanks to BC’s integrated data analytics. They can also observe the change in the data in real-time, allowing them to make real-time choices such as transaction blocking. The authors focused on reducing the computing process at the fog nodes by employing a variety of methods. Smart contracts were established to facilitate the validation of transactions that did not occur at the same time. Authors have paid high attention to performance issue and have come with several designs and strategies to enhance FC performance by managing FC resources ([59, 66, 67, 69, 78, 89, 92, 99, 118, 131, 134, 154, 158, 166, 180, 215]), to decrease latency ([54, 55, 105, 150, 186]), decrease energy consumption ([68, 71, 203]), and decrease the fault tolerance level ([119, 132, 148, 226]). The majority of the selected studies under this category reported that BC can enhance resources’ managing in FC, followed by latency purpose, and the least purpose mentioned was to decrease data consumption.

## Open issues and future trends

Several insights into the limitations of the BC with FC integration and the usefulness of BC across a wide range of purposes may be gained from this SLR. As mentioned in Section 5, BC with FC integration is presently used in a wide range of disciplines and businesses, giving unlimited exploration potential. However, difficulties and obstacles occur, just as they do with any other new technology. We highlight some of the limitations of the BC with FC integration in this part, as well as various options for future research initiatives (Table 3). Because of the FC and BC features, the stated challenges of BC with FC integration have risen. The following challenges of BC with FC integration, as mentioned in Section 2, are mainly based on the Bitcoin BC drawbacks, according to the available literature. While scalability challenge is mainly caused by a lack of FC resources, security, privacy, and standards issues are primarily caused by a lack of BC capabilities and rules. On the other hand, quantum, AI, and big data are affected both BC and FC capabilities. In any of these scenarios, these challenges will have an impact on FC’s performance. As a result, it’s essential to investigate the BC-based challenges that impact FC performance.

**Table 3:** BC with FC integration limitations and future directions

Challenge	Research directions
Scalability [85, 98]	• As a result of the storing of network-wide transactions, the storage needs must be raised • Latency remains an issue due to the huge amount of data processed • Various consensus algorithms consume a lot of energy • Block creation confirmation times are long
Security & Privacy [64, 90, 192, 200, 231]	• In most cases, user data in public BC is available to anybody • Off-chain alternatives are still a source of contention • Privacy problems arise as a result of pseudonymous techniques
Standards and regulations [85, 164, 232]	• It is necessary to create standards for building safe smart contracts that cannot be exploited for harmful reasons • On a worldwide scale, competent and uniform rules and regulations are necessary • Interoperability issue due to different consensus models, transaction methods, and smart contract functionality
Quantum, AI & Big Data [109, 233–236]	• Complex big data analytics techniques on limited resources are a significant problem for FC and BC • Authenticating the training data sets might be a significant challenge • Evaluation and standardization of post-quantum cryptography primitives are required

### Scalability issues

Because IoT devices may create massive amounts of data in real-time, storage capacity and scalability are major concerns with BC with FC integration. Since the underlying BC is ever-expanding, all nodes must keep the whole chain to completely validate any new blocks [210]. Most existing BCs, on the other hand, can only handle a limited number of transactions at once and are not meant to store huge amounts of data; attempting to do so results in significant latency. This, in turn, has an impact on the performance of FC’s limited resources, since it is unable to cope with the massive volume of data generated. To create innovative approaches for simplifying real-time processing and storage, such as data compression and data lightning, a comprehensive description of projected network performance and network scalability is necessary.

The new techniques that tend to decrease latency and optimize BC with FC integration storage, such as off-chain transactions and Sharding, imply some further modifications in the default balance of scalability, security, and decentralization that BCs offer. Therefore, a significant amount of research must be done to find the right balance [98]. Moreover, the use of BC is a crucial element in determining how much energy is consumed in a system. Because the majority of the suggested applications employ a PoW-based BC, despite many attempts to use other algorithms, energy consumption remains an issue. Other algorithms have limitations that some applications cannot tolerate; this may motivate the research community to seek out other substitutions to the PoW algorithms while maintaining the excellent security and dependability that PoW provides [85].

Typically, QoS metrics like latency, energy use, and operating costs are high. The current consensus models are not scalable and frequently fail to deliver satisfactory QoS about throughput and latency, for real-world applications. These two criteria have not been attained at a sufficient QoS level in several current and well-liked public BC systems [237]. For example, while Bitcoin can process 7 transactions per second (TPS), it also experiences a large consensus execution delay time, which can last up to 10 minutes on average [44]. New resource scheduling strategies are required to decrease energy consumption without compromising the quality of service (QoS), including timeliness, dependability, availability, affordability, security, and privacy.

### Security and privacy issues

Despite its many advantages in terms of data security and privacy, BC has several limits and flaws. Because information is kept on a public ledger, privacy and confidentiality remain a challenge for BCs [86]. To safeguard the confidentiality of the data, several anonymization or encryption-based techniques might be used. These methods, however, are not a panacea and are dependent on the system’s implementation and environment. While BC improves FC data flexibility and security, it may have an impact on functions like reliability and data integrity in FC [90]. BC verifies the identity of the data creator and guarantees that the data is immutable and capable of detecting any changes. When data that has already been compromised comes to the BC, however, the system is restricted; it is conceivable that the corruption will not be recognized and the data will stay damaged. Furthermore, data corruption occurs not just as a result of hostile attacks, but also as a result of other factors such as the surrounding environment and device failure [90].

Although all BC systems utilize cryptographic methods to safeguard their data and processes, this does not rule out the possibility of security flaws. Wallet apps are one source of vulnerabilities in Bitcoin that might expose transaction data [200]. Ethereum’s data and contracts are encoded but not encrypted. Ethereum shares many of the same flaws as Bitcoin classic (e.g., weak against 51% attacks). Hyperledger Fabric devotes a significant amount of its protocol to addressing security concerns such as preventing transactions from being connected to users, digital signatures, and access control methods. However, not all of these functions have yet been implemented. To guarantee that communication between all nodes is safe, the Ripple network uses transport layer security. The actual transaction data is encrypted and only the two people involved have access to it. Multichain has an integrated user permissions management system that ensures that only the participants who have been chosen may see the transactions. In the case of a fork, the accountable party may be determined in Eris [185].

There is a trade-off between availability and consistency in BC with FC integration as a distributed architecture of data systems. At the expense of consistency, BC remains accessible and partition tolerant. The BC in Ethereum was found to be much quicker than Bitcoin [231]. Many BC applications require numerous confirmations for newly mined blocks to avoid transactions from double-spending, which is one of the primary consequences of a faster block time. Smart contracts are policy agreements between transactional parties that are not legally enforced by the outside network. Any attacks on smart contracts can put organizations, block miners, and the entire BC network in danger. Based on the foregoing, extensive study is required to secure BC with FC integration [192].

Location and use privacy are two well-known issues in BC data privacy [64]. The traceability of transactions that are propagated through the network is a major concern for most organizations and people [86]. Furthermore, the usage of pseudonyms, for example, is insufficient to maintain transactional privacy. Moreover, Bitcoin transactions have been shown to provide a considerable number of sensitive information. Despite several attempts to remedy the issue, the BC with FC integration location and use privacy issues remain unresolved. More study is needed to develop more privacy methods and techniques [64].

### Regulations and standards

Because BC is such a young and immature technology, there is a lack of standards, which stymies its widespread adoption and delays progress. As described in Section 4, BC has been proposed for usage in a variety of applications. Many nations are also contemplating using BC technology in government contexts including voting, banking, and eHealth [24]. Therefore, there has to be a high level of uniformity across the many parties involved to enable all of these different infrastructures and applications. As more nations choose BC as a solution, the problem of standards and regulations will become even more critical [164]. On the other hand, the Bitcoin BC platform, for example, saves data that can be connected to persons and shared across numerous businesses. As a result, regulations, standards, and social norms must be established to specify how the platform can be used legally and fairly. Moreover, proper enforcement of smart contracts is required to avoid any disputes between transacting organizations. Furthermore, the material saved on BC may include unlawful information, putting the BC in legal jeopardy [185]. Shortage in the regulations will have a direct impact on FC norms like consumer trust and the smoothness of data transmission between IoT devices and FC or between fog nodes.

Cryptocurrencies and the digital economy are built on BC technology. Because bitcoin principles are still not widely understood or legalized in many places, BC technology is unwittingly unlawful. We have shown in this survey how BC can be used for a variety of purposes other than digital money; this information must be shared internationally since BC is different from digital money, but it is the backbone of it. Since such technology is prohibited, it falls behind worldwide technical trends, making it difficult for any BC-based solution to succeed [85]. Although the absence of standards in BC benefits developers, it creates severe communication problems owing to a lack of interoperability. The availability of several BC networks with distinct consensus models, transaction methods, and smart contract features is a big barrier to interoperability. Using existing standards in BC networks is one option for dealing with this problem. Another option is to create new standards. The Enterprise Ethereum Alliance (EEA), for example, has released a standard version of the Ethereum BC [232].

### Quantum resilience, artificial intelligence, and big data analysis

When BC was first conceived, quantum computing did not appear to be particularly close. Recent advances, on the other hand, have caused us to rethink the issue completely. Most BCs, like Bitcoin, employ the SHA-256 hash algorithm which would take a quantum computer 2^128^ operations to crack using Grover’s technique [22]. While SHA-256 is immune to quantum attacks as a result of this, the public key encryption algorithms that most of them utilize are not. After developing a quantum computer and individuals and businesses started using it, the algorithm will be broken, rendering nearly all BCs unsafe. There is now a large effort underway to evaluate and standardize post-quantum cryptography primitives [22]. Despite the efforts made to solve the quantum issue (e.g., [233–236], quantum resilience becomes a serious concern when we construct systems based on BCs that we hope to maintain for many years [238, 239].

Deep learning, along with quicker processors and bigger storage capacities, has cleared the path for modern auditing. Machine learning algorithms, on the other hand, are at the heart of AI and are characterized by their opacity. In this sense, BCs can give auditable trails to show why an AI system made a specific choice and reconcile inconsistencies caused by non-linear usage of many variables and randomization. AI enables a slew of fascinating and creative BC-based applications that might improve the technology’s transparency [22]. The learning process requires a good data sample to create acceptable training data sets. If the adversary is aware of the attack type and has access to the training dataset, the attack type may be readily changed. As a result, understanding the exact nature of an attack to distinguish between desirable and undesired network states is a challenging topic that requires further investigation [109].

The BC structure, which is claimed to be safe and verifiable, may be utilized to make massive data administration easier. Data analyses utilizing the BC structure, on the other hand, entail far very high overhead. Notwithstanding, most cases do not necessitate evaluating all transactions, therefore intermediary or economical supplementary constructs can be developed, increasing overall performance. Despite efforts to introduce big data analysis, traditional big data analysis remains a significant barrier to BC with FC integration [109]. The resources for fog nodes and BC are still limited. Uploading the data to clouds for processing and big data analysis can be a solution, however, this might cause severe latency and privacy issues. Furthermore, anonymized data might make big data analysis difficult to implement, and decrypting data a time-consuming process, resulting in inefficient data analytics. In a nutshell, these new technological developments will have a significant influence on FC performance, making the total integration of BC with FC integration problematic.

## Discussion

The body of knowledge on BC with FC integration is relatively scattered. As a result, this research conducted an SLR and presented a holistic explanation of the purposes of this integration. The purpose of the paper was to address two research questions: How do the purposes of blockchain-fog computing integration develop over time? What are the future challenges in integrating blockchain with fog computing? (RQ2). We evaluated all relevant literature in all reputable databases, including IEEE, Elsevier, Springer, MDPI, Google Scholar, Taylor, Sage, ACM, and Emerald, in order to address the research questions. This section offers an overall evaluation, implications for the findings, and limitations of this study.

Security, privacy, access control, trust management, data management, scalability management, and performance were the seven purpose categories that this study identified and discussed. The whole transaction history is contained in the data in the BC, which is hashed to protect the ledger. As a result, BC can make sure that the devices are connected. The combined monitoring of linked fog nodes in the BC system makes data fabrication nearly impossible. Data is therefore dispersed among a large number of connected fog nodes to safeguard BC. This improves the transaction’s security, integrity, and confidentiality.

Additionally, employing BFC-based apps will make it simpler to spot fraudulent activity because if an attacker modifies the data in a block, the block’s hash value will change and the block will become invalid. Therefore, only authorized users may access data without going through extra checks if, for instance, many business units within a firm may participate in a shared BC that offers a degree of access control. Moreover, the immutability of BC provides the required dependability and confidence for companies among nodes by guaranteeing that a fog node is in control of its identity. As a result, there will be more transparency, which will boost productivity and trust. The integrated data analytics capabilities of BC also enable financial institutions to settle cross-border transactions, particularly those involving significant quantities of money, in almost real-time. They can also see how the data is changing in real-time, which enables them to make decisions like transaction banning in real-time.

While a lack of FC resources mostly contributes to the scaling difficulty, BC design and a lack of regulations may lead to security, privacy, and standards difficulties. On the other hand, both BC and FC capacities are impacted by quantum, AI, and big data. These difficulties will affect FC’s performance in each of these cases. Additionally, the lack of standards provides a challenge to the effectiveness of BC with FC integration; hence, future research and industry efforts must concentrate on developing novel methods and efficient distributed control systems to regulate BC with FC integration. The findings of this study are established in the publicly available literature. Although the use of the Bitcoin platform may have contributed to many challenges and future developments, alternative platforms, such as Ethereum, multichain, and others, should also be looked into. Additionally, since both FC and BC technologies are relatively young, future research and industrial efforts are evolving daily, which makes it challenging to review all data in real-time. The results of this study therefore only applied to the first quarter of 2022. Therefore, future research may use these findings as a foundation and move on from there.

## Conclusions

While FC has gained widespread acceptance as a solution to various cloud computing shortages, many concerns remain unresolved. Many of these difficulties can be addressed by combining FC with BC. BC with FC integration seems to provide more secure, scalable, and efficient applications through the combination of BC and FC capabilities. While BC with FC integration seems reasonable, however, there is a need to provide a synthesized knowledge base of their purposes including challenges for future research directions. We addressed this significant requirements and provided a systematic review and synthesis of recent studies, published in the public domain, with a special emphasis on BC with FC integration purposes. We identified seven major themes of BC with FC integration purposes including security, privacy, access control, trust management, data management, scalability, and performance. Within each of these themes, several purposes were also identified and discussed. These themes and underpinning purposes intend to help academics and practitioners to formulate BC with FC integration strategies for their effective adoption of IoT data handling. Moreover, the critical open research problems, impeding the broad use of BC with FC integration, were also identified and reported in this paper. By offering major advances in terms of security, privacy, data management, and trust management, it is anticipated that BC can restructure and revolutionize the future of FC technology. However, BC with FC integration raises several technological issues, including scalability, a lack of standards and regulations, quantum resilience, and AI advancement, which could be further explored in future research studies.

## Data Availability

Not applicable.
